# The Integrative Analysis of microRNA and mRNA Expression in Mouse Uterus under Delayed Implantation and Activation

**DOI:** 10.1371/journal.pone.0015513

**Published:** 2010-11-29

**Authors:** Ren-Wei Su, Wei Lei, Ji-Long Liu, Zhi-Rong Zhang, Bo Jia, Xu-Hui Feng, Gang Ren, Shi-Jun Hu, Zeng-Ming Yang

**Affiliations:** 1 Key Laboratory of the Ministry of Education for Cell Biology and Tumor Cell Engineering, School of Life Science, Xiamen University, Xiamen, China; 2 School of Life Science, Northeast Agricultural University, Harbin, China; Institute of Zoology, Chinese Academy of Sciences, China

## Abstract

**Background:**

Delayed implantation is a developmental arrest at the blastocyst stage and a good model for embryo implantation. MicroRNAs (miRNAs) have been shown to be involved in mouse embryo implantation through regulating uterine gene expression. This study was to have an integrative analysis on global miRNA and mRNA expression in mouse uterus under delayed implantation and activation through Illumina sequencing.

**Methodology/Principal Findings:**

By deep sequencing and analysis, we found that there are 20 miRNAs up-regulated and 42 miRNAs down-regulated at least 1.2 folds, and 268 genes up-regulated and 295 genes down-regulated at least 2 folds under activation compared to delayed implantation, respectively. Many different forms of editing in mature miRNAs are detected. The percentage of editing at positions 4 and 5 of mature miRNAs is significantly higher under delayed implantation than under activation. Although the number of miR-21 reference sequence under activation is slightly lower than that under delayed implantation, the total level of miR-21 under activation is higher than that under delayed implantation. Six novel miRNAs are predicted and confirmed. The target genes of significantly up-regulated miRNAs under activation are significantly enriched.

**Conclusions:**

miRNA and mRNA expression patterns are closely related. The target genes of up-regulated miRNAs are significantly enriched. A high level of editing at positions 4 and 5 of mature miRNAs is detected under delayed implantation than under activation. Our data should be valuable for future study on delayed implantation.

## Introduction

Embryo implantation is a mutual interaction between blastocyst and uterus. The successful implantation of an embryo is dependent on both proper preparation of active blastocyst and receptive endometrium [1]. Delayed implantation is a developmental arrest at the blastocyst stage and a good model for deciphering the molecular interaction between embryo and uterus. There are around 100 species of mammals undergoing delayed implantation [2]. Because estrogen is essential for on-time uterine receptivity and blastocyst activation in mice [3], ovariectomy on day 4 of pregnancy will lead to blastocyst dormancy [4]. Many specific factors have been identified to be essential for embryo implantation through large-throughput analysis [5], [6], and global gene expression in mouse uterus during delayed implantation and activation was also examined by Reese et al [5]. The global gene expression in mouse blastocysts during delayed implantation and activation was also reported [4]. However, the mechanism underlying delayed implantation and activation is still unclear.

Except for protein-coding RNAs, microRNAs (miRNAs) have been shown to be involved in mouse embryo implantation through regulating uterine gene expression [7], [8]. Extensive sequence variations (isomiRs) for almost all miRNA and miRNA* species add additional complexity to the miRNA transcriptome [9]. RNA editing from A to I is widely present in human [10], [11]. Additionally, this kind of editing was also detected in the seed sequences of miRNAs and may have effects on the recognition of target genes [11]. Illumina sequencing has opened the door for detecting and profiling known and novel miRNAs and mRNAs at unprecedented sensitivity. These latest high-throughput strategies permit high-resolution views of expressed miRNAs over a wide dynamic range of expression levels [9]. Direct sequencing also offers the potential to detect variations in mature miRNA length, as well as enzymatic modifications of miRNAs [12].

The large-scale proteomic analysis in mouse uterus during embryo implantation is still lacking. Because miRNAs can down-regulate some of their targets not only at the translational but also at the transcriptional level [13], and the expression profiles of intragenic miRNAs and of their corresponding host genes are very similar both at the tissue and cellular level [14], [15], it is therefore possible to use the paired expression analysis of miRNAs and mRNAs to identify mRNA targets of miRNAs. Serial analysis of gene expression (SAGE) is a high-throughput method for global gene expression analysis that allows the quantitative and simultaneous analysis of a large number of transcripts [16]. Therefore, the combination of SAGE and Illumina sequencing seems to be perfectly suited for deep transcriptome analysis [17].

This study was to have an integrative analysis on global miRNA and mRNA expression in mouse uterus under delayed implantation and activation through Illumina sequencing. We found that miRNA and mRNA expression patterns are closely related. A higher level of editing at positions 4 and 5 of mature miRNAs is detected under delayed implantation than under activation. The data from this study would provide a combined and comprehensive tissue-specific analysis of diverse miRNAs and transcriptional activity and also shed new light into the fine-tuning process of implantation.

## Results

### Illumina sequencing of small RNAs

Total RNAs from mouse uteri under delayed implantation and activation were used to construct small RNA libraries for sequencing. The raw data from Illumina sequencing is available at Gene Expression Omnibus (GEO: GSE19473). In our two libraries, there were 5,334,521 reads for delayed implantation and 5,618,688 reads for activation, respectively. The read size was mainly ranged from 21 to 23 nt. The percentage of the 22 nt reads in total reads was 53.17% for delayed implantation and 54.10% for activation, respectively.

The most abundant (based on read count) RNA species in both libraries were classified as miRNAs, representing 74.52% of delayed implantation library and 76.57% of activation library, respectively (Table 1). A high percentage of small RNAs were sorted as unknown RNAs, 23.78% for delayed implantation and 21.34% for activation. There were small amounts of piRNAs (≤0.22%), tRNAs (≤0.22%), rRNAs (≤0.26%), snRNAs (≤0.01%), snoRNAs (≤0.24%), mRNAs (≤0.47%) and genomic RNAs (≤0.84%).

**Table 1:** The category of small RNAs.

Category	Delay	Delay (%)	Activation	Activation (%)
miRNA	3,972,448	74.52	4,302,233	76.57
piRNA	11,912	0.22	8,565	0.15
tRNA	9,900	0.19	12,345	0.22
rRNA	9,377	0.18	14,823	0.26
snRNA	434	0.01	525	0.01
snoRNA	12,875	0.24	7,847	0.14
mRNA	18,321	0.34	26,607	0.47
genomic	30,611	0.57	46,954	0.84
unknown	1,268,643	23.78	1,198,789	21.34
Total	5,334,521	100	5,618,688	100

In both libraries, the top 5 most abundant miRNAs were let-7c, let-7f, let-7a, let-7b and miR-199b, representing 71.18% for delayed implantation and 71.10% for activation among total miRNAs (Table S1). Let-7c was the most abundant miRNA in both libraries, 33.62% for delayed implantation and 31.71% for activation, respectively.

Read counts from delayed and activated uterus were normalized to tags per million (TPM) for each library. Differentially-expressed miRNAs were selected according to their fold changes (>1.2 fold), TPM of either library (>100) and p-values (<0.001). Based on the above-mentioned standards, there were 20 miRNAs up-regulated (Table 2) and 42 miRNAs down-regulated (Table 3). There were three up-regulated miRNA* sequences detected, including miR-17*, miR-145* and miR-21*.

**Table 2:** Significantly up-regulated miRNAs.

miRNA name	Activation(TPM)	Delay(TPM)	folds(Activation/Delay)	p value
mmu-miR-423-5p	4,273	3,452	1.24	0
mmu-miR-221	1,095	868	1.26	0
mmu-miR-17*	239	188	1.27	1.06E-05
mmu-let-7f	128,615	101,036	1.27	0
mmu-miR-320	13,741	10,754	1.28	0
mmu-let-7d	24,228	18,731	1.29	0
mmu-miR-98	334	257	1.30	5.40E-10
mmu-miR-345-3p	274	210	1.30	4.08E-08
mmu-miR-128	227	169	1.35	2.18E-08
mmu-miR-145*	106	78	1.37	8.11E-04
mmu-miR-21	14,877	10,297	1.44	0
mmu-miR-33	108	63	1.71	1.34E-11
mmu-miR-341	127	70	1.81	0
mmu-miR-92a	1,411	733	1.93	0
mmu-miR-298	329	130	2.53	0
mmu-miR-134	207	80	2.60	0
mmu-miR-21*	152	52	2.91	0
mmu-miR-7a	102	30	3.42	0
mmu-miR-146b	8,297	2,355	3.52	0
mmu-miR-805	437	118	3.71	0

Note: TPM>100, fold>1.2, p<0.01.

**Table 3:** Significantly down-regulated miRNAs.

miRNA name	Activation(TPM)	Delay(TPM)	Folds (Activation/Delay)	p value
mmu-miR-145	353	915	−2.56	1.29E-268
mmu-miR-429	116	260	−2.27	3.42E-59
mmu-miR-138	106	225	−2.13	8.45E-46
mmu-miR-31	247	491	−2.00	1.05E-86
mmu-miR-23b	675	1,321	−1.96	2.49E-224
mmu-miR-652	58	109	−1.85	9.09E-16
mmu-miR-29c	253	466	−1.85	2.91E-67
mmu-miR-200a	342	603	−1.75	4.54E-77
mmu-miR-15a	98	170	−1.72	3.62E-20
mmu-miR-200b	186	319	−1.72	6.38E-37
mmu-miR-16	990	1,681	−1.69	2.56E-191
mmu-miR-23a	1,290	2,178	−1.69	4.83E-244
mmu-miR-322	100	168	−1.67	7.51E-18
mmu-miR-214	177	289	−1.64	1.95E-28
mmu-miR-196b	768	1,242	−1.61	2.85E-119
mmu-miR-15b	124	201	−1.61	9.50E-19
mmu-miR-29b	357	534	−1.49	3.77E-37
mmu-miR-181c	98	146	−1.49	3.25E-09
mmu-miR-99a	625	928	−1.49	5.07E-63
mmu-miR-374	74	109	−1.47	2.75E-06
mmu-miR-29a	18,049	26,525	−1.47	0
mmu-miR-26a	7,686	11,285	−1.47	0
mmu-miR-455	86	125	−1.45	4.84E-07
mmu-miR-210	72	106	−1.45	8.49E-06
mmu-miR-195	704	1,023	−1.45	1.40E-62
mmu-miR-126-5p	83	120	−1.45	1.44E-06
mmu-miR-10a	1,839	2,666	−1.45	3.88E-162
mmu-miR-24	7,121	10,058	−1.41	0
mmu-miR-100	103	141	−1.37	1.70E-05
mmu-miR-101a	1,251	1,711	−1.37	6.40E-76
mmu-miR-106b	252	344	−1.37	2.78E-14
mmu-miR-93	307	411	−1.33	1.88E-15
mmu-miR-125b-5p	866	1,158	−1.33	1.46E-44
mmu-miR-181a	3,579	4,767	−1.33	5.76E-181
mmu-miR-200c	547	728	−1.33	1.19E-26
mmu-miR-497	522	692	−1.33	9.59E-25
mmu-miR-10b	389	506	−1.30	1.15E-15
mmu-miR-674	256	327	−1.28	1.57E-08
mmu-miR-22	261	328	−1.25	2.49E-07
mmu-miR-34c	233	284	−1.22	1.45E-04
mmu-miR-30a	4,176	5,033	−1.20	4.98E-85
mmu-let-7b	94,695	113,963	−1.20	0

Note: TPM>100, fold>1.2, p<0.01.

### Editing of mature miRNAs

**Figure 1:**
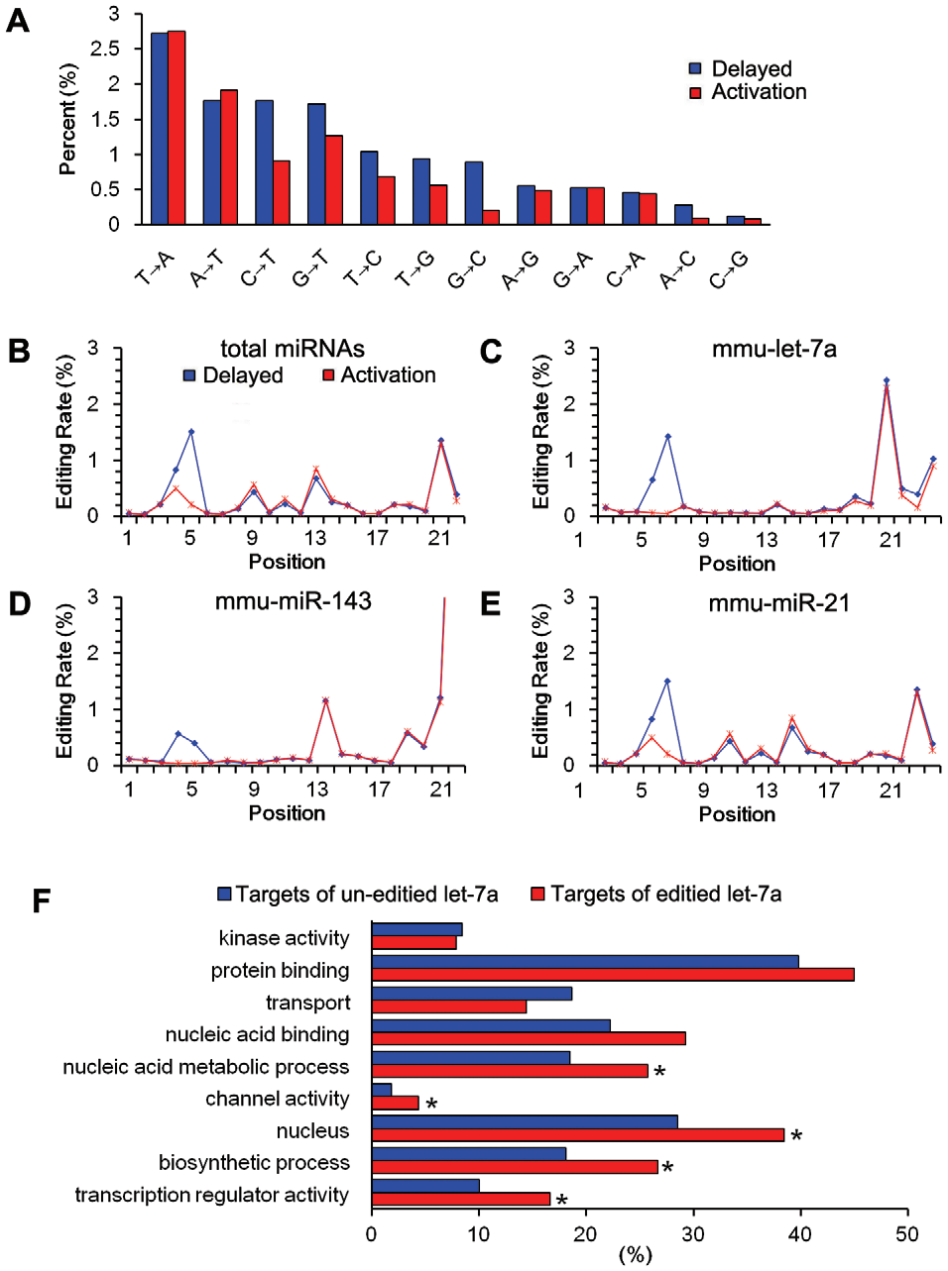
miRNA editing. (A)The percentages of various edited forms in all of the miRNAs. The editing rate of each position of the total miRNAs (B), let-7a (C), miR-143 (D) and miR-21 (E) in mouse uteri from delayed implantation and activation are shown. (F) Gene ontology analysis of the targets of unedited and edited let-7a. The “biological process” of the target mRNAs is compared between two groups. A p value is assigned to each GO term by chi-square test.

There were different forms of editing in both libraries. T to A was the most dominant form in both libraries (2.75% for activation and 2.72% for delayed implantation), followed by A to T, C to T, G to T, T to C, T to G and G to C. Although A to G editing was a prevailing modification in some cell types [18], [19], it was the eighth dominant editing form in total editing, representing 0.48% for activation and 0.55% for delayed implantation (Fig. 1A).

In both libraries, different kinds of nucleotide modification (designated as isoforms) were detected in mature miRNAs. The reference sequence in miRBase was not always the most dominant form in our study. In general, the editing in all of the miRNAs mainly occurred at positions 4, 5, 15, 19, and 21. The percentage of editing at positions 4 and 5 was significantly higher under delayed implantation than that under activation (z test, p<0.01). There was no difference between two libraries for the editing at other positions (Fig. 1B).

For let-7a, the editing rate at position 5 under delayed implantation was significantly higher than that under activation (Fig. 1C). Although the editing rate was very high at position 19, there was no difference between two libraries. Nucleotides at positions 4–5 were located right in the middle of the seed region (nucleotides 2–8) that is important for miRNA-mRNA binding. Such kind of nucleotide modification may alter genuine targets of let-7a.

For miR-143, there was a high percentage of editing at positions 4, 5, 13 and at 3′-end (Fig. 1D). The editing rate at positions 4 and 5 in delayed implantation was also significantly higher than activation. However, the editing rate at position 13 and 3′-end was similar between two libraries. Compared with both miR-143 and let-7a, the editing in miR-21 occurred in more positions, mainly at positions 4, 5, 9, 11, 13, 18, 19 and 21. But the percentage of editing at positions 4 and 5 under delayed implantation was also significantly higher than that under activation group, similar to let-7a and miR-143. (Fig. 1E).

Because the position 5 was located in the middle of seed sequence and the editing rate at position 5 of let-7a was significantly higher in delayed implantation than activation, TargetScan 4.2 (http://www.targetscan.org/) was used to predict target genes to see whether editing would affect the target genes. Only conserved binding sites (conserved in mouse, human, rat and dog) were considered. There were 614 target genes predicted for let-7a unedited form, while 236 target genes for edited form. There are only 27 target genes shared by both unedited and edited forms, suggesting that editing does change the genuine targets of let-7a.

Based on Gene Ontology analysis of the target genes of unedited and edited let-7a, the genes involved in “biological process” were compared between two groups. Compared to unedited let-7a, there were significantly more genes involved in nucleic acid metabolic process, channel activity, nucleus, biosynthetic process and transcription regulator activity in edited let-7a targets (Fig. 1F).

Free energy changes between the wild type let-7a targets and edited let-7a targets were calculated by RNAeval algorithm implemented in the Vienna RNA package. When let-7a was edited from G to C at position 5, the binding between edited let-7a-5C and its target resulted in a net decrease of −4.5 kcal/mol in free energy. However, the free energy of binding between unedited let-7a and its targets was also −6.6 kcal/mol less than the binding between let-7a-5C and the targets of let-7a reference sequence.

All of the let-7a edited isoforms were listed in Table 4. The let-7a reference sequence was the dominant form, representing 56.1% of the total isoforms in delayed uterus. However, the most dominant form of miR-143 was one nucleotide deletion from 3′-end. miR-143 reference sequence was the second dominant form, representing 31.41% of total miR-143 isoforms (Table 5).

**Table 4:** The top 30 editing forms of let-7a.

Activation(TPM)	Delay(TPM)	Mature let-7a sequence
		--UGAGGUAGUAGGUUGUAUAGUU----
103,770	83,751	--TGAGGTAGTAGGTTGTATAGTT----
18,784	17,258	--TGAGGTAGTAGGTTGTATAGT-----
16,922	15,990	--TGAGGTAGTAGGTTGTATAGTTT---
14,662	13,678	--TGAGGTAGTAGGTTGTATAGTTa---
2,742	2,466	--TGAGGTAGTAGGTTGTATAG------
1,996	1,907	--TGAGGTAGTAGGTTGTATtGTT----
928	1,265	--TGAGGTAGTAGGTTGTAT-GTTT---
785	817	--TGAGGTAGTAGGTTGTATAGTa----
921	644	--TGAGGTAGTAGGTTGTATAGTTaa--
636	694	--TGAGGTAGTAGGTTGTAT--------
663	609	--TGAGGTAGTAGGTTGTATA-------
521	532	--TGAGGTAGTAGGTTGTATtGTTT---
509	498	--TGAGGTAGTAGGTTGTATAGTTg---
502	443	--TGAGGTAGTAGGTTGTATAGTaa---
536	367	--TGAGGTAGTAGGTTGTATtGT-----
350	483	--TGAGGTAGTAGGTTGTAT-GTT----
456	368	--TGAGGTAGTAGGTTGTATAGTTTT--
420	318	--TGAGGTAGTAGGTTGTATAGTTaT--
354	348	--TGAGGTAGTAGGTTGTATtGTTa---
6	667	--TGAGcTAGTAGGTTGTATAGTT----
288	305	--TGAGGTAGTAGGTTGTATAtTT----
245	329	--TGAGGTAGTAGGTTGTAT-GTTTT--
247	306	--TGAGGTAGTAGGTTGTAT-GTTTa--
298	226	---GAGGTAGTAGGTTGTATAGTT----
246	190	--TGAGGTAGTAGtTTGTATAGTT----
213	149	--TGAGGTAGTAGGTTGTATAGTTTa--
174	169	--TGAGGTAGTAGGTTGTAcAGTT----
146	161	--TGAGGTAGTAGGTTGTATAGa-----
159	137	--TGAGGTAGTAGGTTGTATAGTaT---
141	148	--TGAGGTAGTAGGTTGTATAa------

Note: The copy number of each read is shown on the left. The canonical mature let-7a sequence (reference sequence) is in the top row. The potential modifications sites were in lower case.

**Table 5:** The top 30 editing forms of miR-143.

Activation(TPM)	Delay(TPM)	Mature miR-143 sequence
		--UGAGAUGAAGCACUGUAGCUC--
6,983	7,124	--TGAGATGAAGCACTGTAGCT---
6,775	6,522	--TGAGATGAAGCACTGTAGCTC--
1,463	1,561	--TGAGATGAAGCACTGTAGCTCt-
1,180	1,191	--TGAGATGAAGCACTGTAGC----
659	794	--TGAGATGAAGCACTGTAGCTa--
319	316	--TGAGATGAAGCACTGTAGCTCA-
302	271	--TGAGATGAAGCACTGTAGCTt--
228	239	--TGAGATGAAGCACTGTAG-----
172	208	--TGAGATGAAGCACTGTAGCTCtt
142	148	--TGAGATGAAGCACTGTAGCTaA-
103	109	--TGAGATGAAGCACTGTAGCTat-
105	106	--TGAGATGAAGCAtTGTAGCT---
89	106	--TGAGATGAAGCACTGTAGCa---
78	84	--TGAGATGAAGCACTGTAGC----
71	67	--TGAGATGAAGCAtTGTAGCTC--
66	56	--TGAGATGAAGCACTGTAGCTtt-
50	64	--TGAGATGAAGCACTGTAGCTCAa
53	55	--TGAGATGAAGCACTGTAGa----
39	39	-CTGAGATGAAGCACTGTAGCT---
32	43	--TGAGATGAAGCACTGTAa-----
35	30	--TGAGATGAAGCACTGTAt-----
27	34	--TGAGATGAAGCACTGTAGCTCAt
29	30	--TGAGATGAAGCACTGTAGCTCTaa
25	28	--TGAGATGAAGCACTGTAGCat---
27	23	--TGAGATGAAGCACTGTAGCTaAa-
25	23	-CTGAGATGAAGCACTGTAGC-----
24	24	--TGAGATGAAGCACTGTAGCTCta-
24	19	--TGAGATGAAGCACTGTAGCTCttt
20	23	TCTGAGATGAAGCACTGTAGCT----
24	16	--TGAGATGAAGCACTGTAGCTttt-

Note: The copy number of each read is shown on the left. The canonical mature miR-143 sequence (reference sequence) is in the top row. The potential modifications sites were in lower case.

For miR-21, the reference sequence was the most abundant sequence. The number of tags at activation was slightly lower (6,756/7,054) than that of delayed implantation. However, the second most abundant sequence having one C addition at 3′-end was significantly higher at activation than delayed implantation (8,427/2,313).The third most abundant sequence was one “A” deletion from 3′-end, and the number of tags at delayed implantation was also slightly higher than that of activation. Because these three sequences were the same from positions 1 to 21 except for 3′ deletion or addition, these sequences should be together detected by reference probe using Northern blot. If the total number of these three sequences were calculated, the tag number at activation was significantly higher than that of delayed implantation (18,964/13,581). The major difference between delayed implantation and activation was derived from the second sequence TAGCTTATCAGACTGATGTTGAC (Table 6).

**Table 6:** The top 30 editing forms of miR-21.

Activation(TPM)	Delay(TPM)	Mature miR-21 sequence
		--UAGCUUAUCAGACUGAUGUUGA---
6,756	7,054	--TAGCTTATCAGACTGATGTTGA---
8,427	2,313	--TAGCTTATCAGACTGATGTTGAC--
3,781	4,214	--TAGCTTATCAGACTGATGTTG----
287	240	--TAGCTTATCAGACTGATGTT-----
359	125	--TAGCTTATCAGACTGATGTTGACa-
367	103	--TAGCTTATCAGACTGATGTTGACT-
180	177	--TAGCTTATCAGACTGATGTTGAa--
171	118	--TAGCTTATCAGACTGATG-------
178	56	--TAGCTTATCAGACTGATGTTtAC--
115	89	--TAGCTTATCAGACTGATGT------
94	80	--TAGCTTATCAGACTGATGTTGAaa-
69	32	-ATAGCTTATCAGACTGATGTTGA---
43	54	-ATAGCTTATCAGACTGATGTTG----
46	38	--TAGCTTATCAGACTGATGTTt----
45	31	--TAGCTTATCAGACTGATGTTGACg-
59	13	--TAGCTTATtAGACTGATGTTGAC--
61	11	--TAGCTTATCAGACTGATGTTGACaa
34	31	--TAGCTTATCAGAtTGATGTTGA---
39	21	--TAGCTTATCAGACTGATGTTGAt--
24	34	--TAGCTTATCAGACTGATGTTtA---
27	30	--TAGCTTATtAGACTGATGTTGA---
52	5	--TAGCTTATCAGACTGATGTTGACc-
47	9	--TAGCTTATCAGAtTGATGTTGAC--
25	30	--TAGCTTATCAGACTGATGTTGt---
41	10	--TAGtTTATCAGACTGATGTTGAC--
3	45	--TAGCcTATCAGACTGATGTTGA---
35	11	---AGCTTATCAGACTGATGTTGAC--
7	37	--TAGCgTATCAGACTGATGTTGA---
22	22	--TAGtTTATCAGACTGATGTTGA---
15	24	--AGCTTATCAGACTGATGTTGA----

The copy number of each read is shown on the left. The canonical mature miR-21 sequence (reference sequence) is in the top row. The potential modifications sites were in lower case.

In this study, the reference sequence of miR-21 at activation was slightly lower (6,756/7,054) than at delayed implantation. Because qRT-PCR was only designed for reference sequence of each miRNA, we used qRT-PCR to confirm whether miR-21 reference sequence was down-regulated in mouse uterus at activation. We found that the level of miR-21 at activation was indeed lower than that at delayed implantation. The miR-21 level in mouse uterus on day 5 of pregnancy was also checked by qRT-PCR. The level of miR-21 at implantation site was also lower than that at inter-implantation sites (data not shown).

### Target genes of edited let-7a

**Figure 2:**
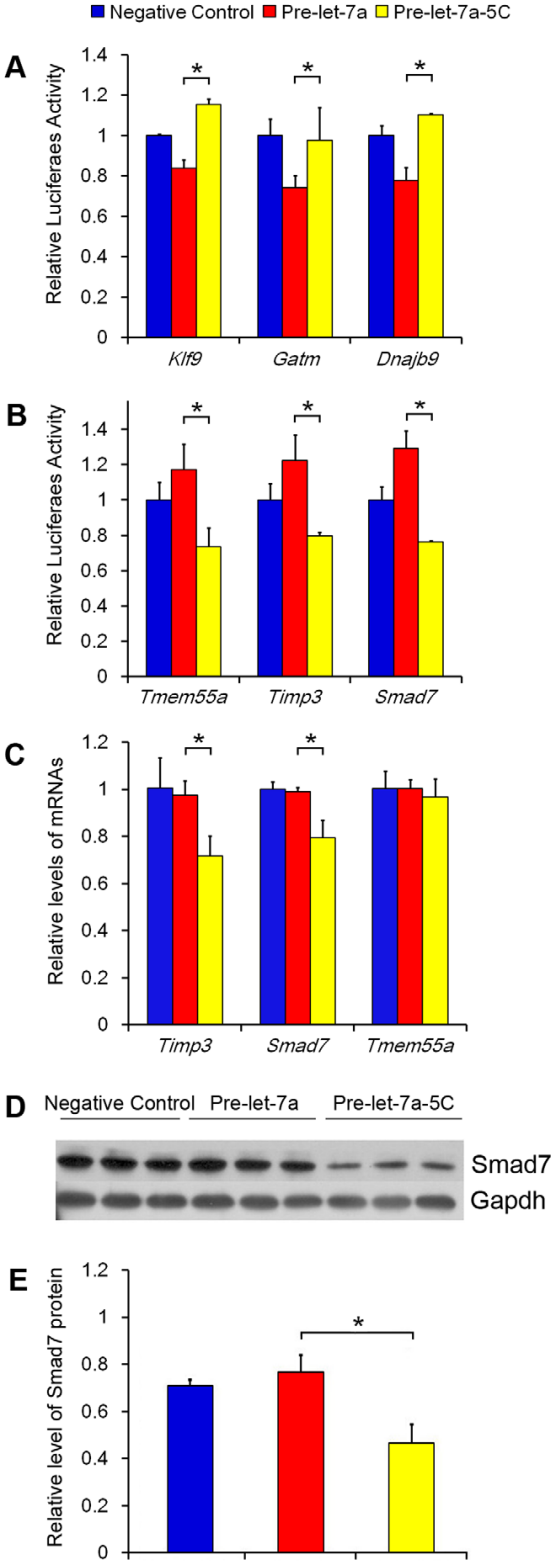
Prediction and confirmation of the target genes predicted for both let-7a (unedited) and let-7a-5C (edited). (A) The confirmation of the target genes (*Klf9*, *Gatm* and *Dnajb9*) of let-7a in mouse 3T3 cells using dual-luciferase assay. Cells were co-transfected with negative control, let-7a (Pre-let-7a) or let-7a-5C (Pre-let-7a-5C) precursor, respectively; (B) The confirmation of the target genes (*Tmem55a*, *Timp3* and *Smad7*) of let-7a-5C in mouse 3T3 cells; (C) The relative mRNA expression level of the three target genes of edited let-7a-5C in cultured mouse stromal cells transfected with negative control, Pre-let-7a or Pre-let-7a-5C; (D) Protein level of Smad7 was detected by Western blot after mouse stromal cells were transfected with negative control, Pre-let-7a or Pre-let-7a-5C; (E) Quantification of Smad7 protein expression in (D). The difference between Pre-let-7a and Pre-let-7a-5C was compared using t-test, and the significant difference between two groups was labeled with asterisk.

Compared to delayed implantation, let-7a was significantly up-regulated under activation. Furthermore, G to C editing at position 5 was significantly higher under delayed implantation than under activation (667/6). Because G to C editing at position 5 was just located in the middle of the binding sequence ‘seed sequence’, further experiment was performed to examine whether this editing would shift the direction of targeting. *Klf9, Gatm* and *Dnajb9* were predicted to be the target genes of unedited let-7a, whereas *Tmem55a*, *Timp3* and *Smad7* were predicted to be target genes of edited let-7a-5C. To confirm that these predicted target genes were indeed the target gene of their corresponding miRNAs, the 3′-UTR segment of each gene was amplified by PCR from mouse cDNA and inserted into downstream of the luciferase reporter gene in the pGL3 control vector for the Dual-Luciferase assay. Compared to negative control, the luciferase activity containing 3′-UTR of *Klf9*, *Gatm* and *Dnajb9* was significantly inhibited by transfection with let-7a precursor, respectively (Fig. 2A). Similarly, the luciferase activity containing 3′-UTR of *Tmem55a*, *Timp3* and *Smad7* was also significantly inhibited by transfection with edited let-7a-5C precursor (Fig. 2B). Furthermore, compared to let-7a-5C precursor, the luciferase activity containing 3′-UTR of *Klf9*, *Gatm* and *Dnajb9* was significantly inhibited by transfection with let-7a precursor, respectively (Fig. 2A). Conversely, compared to let-7a precursor, the luciferase activity containing 3′-UTR of *Tmem55a*, *Timp3* and *Smad7* was significantly inhibited by transfection with edited let-7a-5C precursor (Fig. 2B).

In order to examine whether let-7a-5C could regulate its target genes in cultured mouse uterine cells, mouse uterine stromal cells were transfected with let-7a-5C precursor or let-7a precursor and cultured for 24 h. The expression of *Tmem55a*, *Timp3* and *Smad7* was determined by qRT-PCR. Compared to let-7a precursor, both *Timp3* and *Smad7* were significantly inhibited by let-7a-5C precursor (Fig. 2C). Additionally, Smad7 protein was significantly inhibited by let-7a-5C in comparison with let-7a precursor (Fig. 2D, E). There was no detectable change for *Tmem55a* between let-7a and let-7a-5C treatments (Fig. 2D).

**Figure 3:**
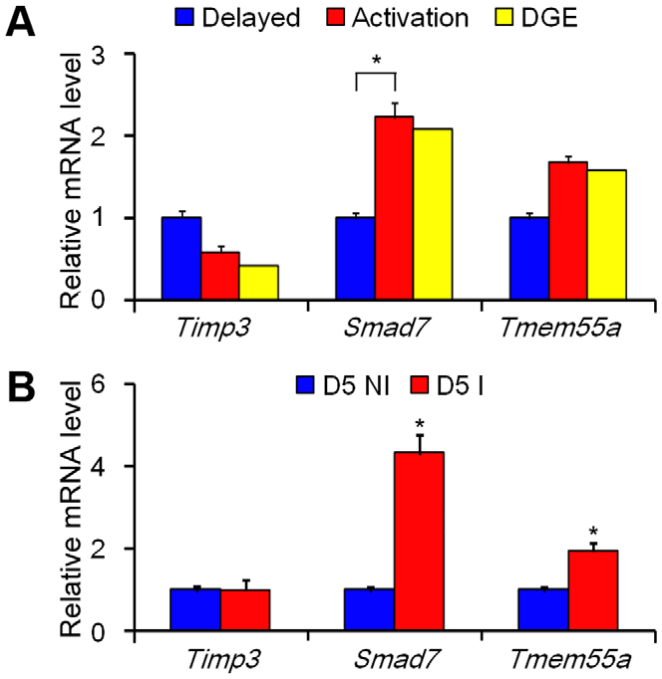
qRT-PCR analysis of the mRNA level of three target genes of let-7a-5C in mouse uteri. (A) The mRNA level of each gene in mouse uterus under delayed implantation and activation. The data from SAGE-Illumina sequencing (DGE) in mouse uterus under delayed implantation and activation were marked in yellow color; (B) The mRNA level of each gene in mouse uterus on day 5 of pregnancy at implantation sites (D5 I) and non-implantation sites (D5 NI).

Because let-7a-5C was significantly up-regulated under delayed implantation, we checked our SAGE-Illumina sequencing data to examine whether there was a reverse relation between let-7a-5C and its three target genes. In our SAGE-Illumina sequencing data, both *Smad7* and *Tmem55a* were up-regulated under activation, which was opposite to the expression of let-7a-5C (Fig. 3A). When qRT-PCR was used to measure the expression of three target genes in mouse uterus, both *Smad7* and *Tmem55a* were also up-regulated under activation (Fig. 3A). However, *Timp3* was down-regulated under activation in our SAGE-Illumina sequencing data, which was also confirmed by qRT-PCR (Fig. 3A). Our data suggest that *Timp3* may be not regulated by let-7a-5C at least at the level of mRNA expression in mouse uterus.

In order to examine whether the expression of *Tmem55a*, *Timp3* and *Smad7* was similar between activation of delayed implantation and implantation sites, the expression of *Tmem55a*, *Timp3* and *Smad7* was also examined in mouse uterus on day 5 of pregnancy. Compared to inter-implantation sites, both *Smad7* and *Tmem55a* were also highly expressed at implantation sites. However, *Timp3* expression didn't significantly change between implantation and inter-implantation sites (Fig. 3B).

**Figure 4:**
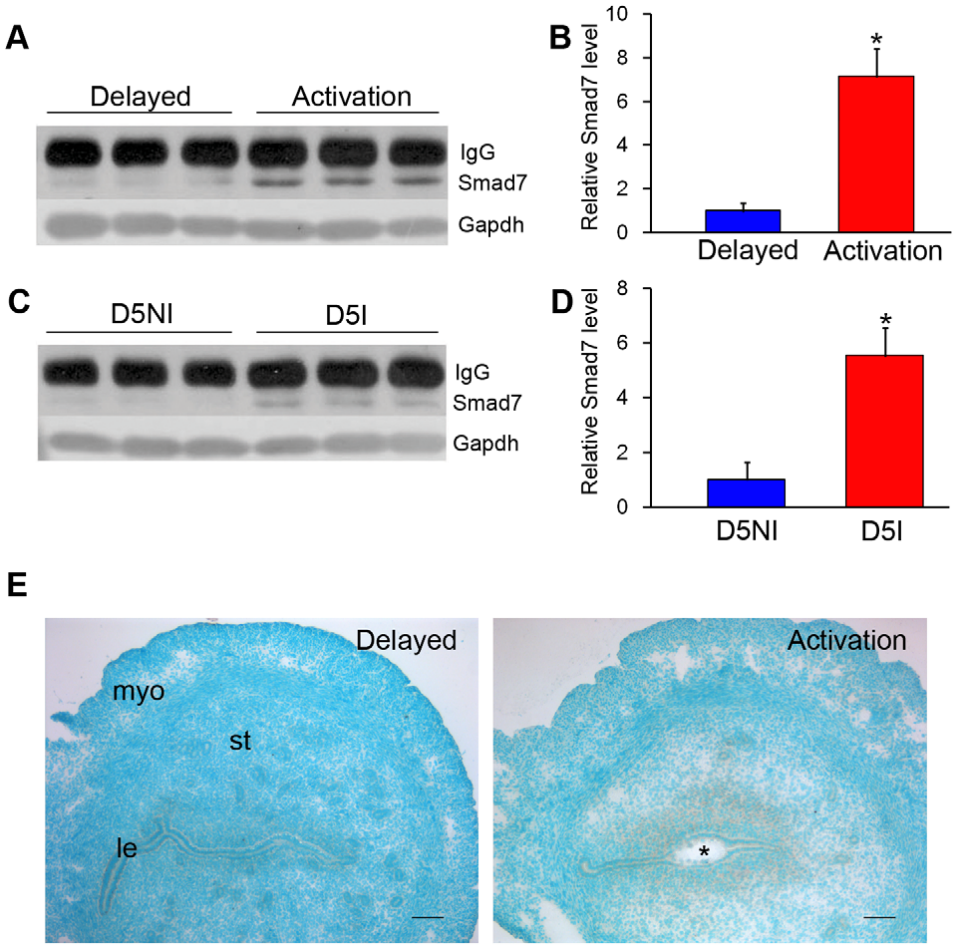
Smad7 expression in mouse uterus. (A) Smad7 protein under delayed implantation and activation by Western blot; (B) Quantification from data in (A); (C) Smad7 protein in mouse uterus on day 5 of pregnancy by Western blot; (D) Quantification from the data in (C); (E) In situ hybridization of Smad7 mRNA expression in mouse uterus under delayed implantation (Delay) and activation. le: luminal epithelium; myo: myometrium; st: Stroma; *: the implanting blastocyst. Bar = 60 µm.

Because *Smad7* was verified as a target gene of let-7a-5C, its protein level was also examined by Western blot. Compared to delayed implantation, Smad7 protein was up-regulated under activation (Fig. 4A, B). The level of Smad7 protein at implantation sites was also stronger than that at inter-implantation sites (Fig. 4C, D). By in situ hybridization, *Smad7* mRNA expression was mainly localized in the subluminal stromal cells under activation of delayed implantation, but no *Smad7* signal was detected in the uterus under delayed implantation (Fig. 4E).

### Novel miRNAs

In our study, a high percentage of small RNAs were sorted as unknown RNAs, 23.78% for delayed implantation and 21.34% for activation. We would like to check whether novel miRNAs were present among unknown RNAs. Novel miRNAs were predicted by miRDeep. The miRDeep cut-off score was set at 1. Based on miRDeep software, 6 novel miRNAs were predicted. The total tag number for all of 6 novel miRNAs in both delayed implantation and activation was 538, accounting for 0.02% of the unknown RNAs and 0.005% of the total RNAs. Compared to mature miRNA strand, the percentage of miRNA* sequences was significantly less. For the miRNA* sequences of the novel miRNAs, there were 7 tags for delayed implantation and 22 tags for activation. The number of the tags for nov-miRNA-4 was 75 for delayed implantation and 76 for activation (Table 7). Since these novel miRNAs were expressed at a very low level in mouse uterus, whether these novel miRNAs are functional is still unknown.

**Table 7:** List of novel miRNAs.

ID	Tags (del+act)	Mature sequence
nov-miR-1	155 (101+54)	ggggugugcucagagcagguggccu
nov-miR-2	24 (4+20)	acccgucccguucguccccgga
nov-miR-3	15 (8+7)	aggggagcuagguagaaagcca
nov-miR-4	151 (75+76)	auuggaguucaugcaaguucu
nov-miR-5	16 (11+5)	cccuggaaggagacguggauuc
nov-miR-6	177 (70+107)	cuaaggcaggcagacuucagugu

Note: Novel miRNAs were predicted by miRDeep. The miRDeep cut-off score was set at 1.

**Figure 5:**
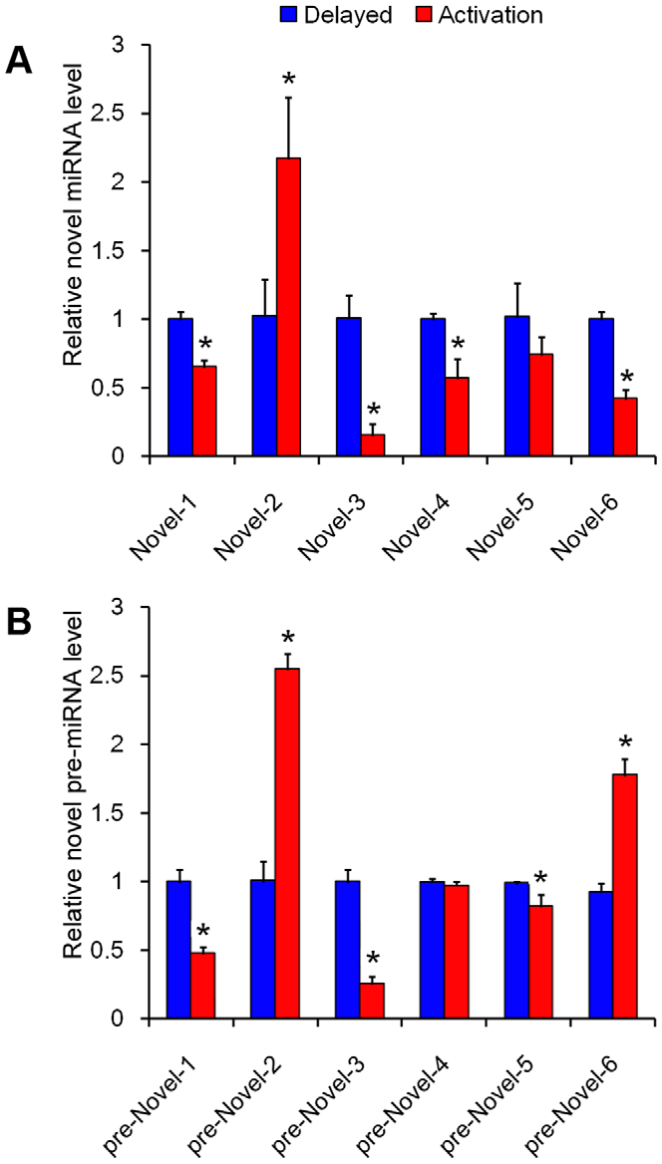
Prediction and confirmation of the novel miRNAs. (A) qRT-PCR of mature miRNAs; (B) qRT-PCR of precursor miRNAs.

Because novel mouse miRNAs were expressed in the delayed and activated uterus, qRT-PCR was used to verify their expression in mouse uterus. For novel miRNAs, primers were synthesized according to the paper previously published with some modifications [19]. Compared to delayed implantation, nov-miRNA-1, nov-miRNA-3, nov-miRNA-4, and nov-miRNA-6 were significantly down-regulated in mouse uterus under estrogen activation, whereas nov-miRNA-2 was significantly up-regulated under estrogen activation. The expression of nov-miRNA-5 was not significantly different between delayed implantation and activation (Fig. 5B).

In order to examine whether these novel miRNAs were co-transcribed with their precursors, the expression of their precursors was also measured. The primers for their precursors were designed based on the pre-miRNA sequences predicted by miRDeep software. The expression trend of their precursors was very similar to that of their corresponding miRNAs, except for pre-miR-6, which was significantly up-regulated under activation (Fig. 5C).

### Digital gene expression from SAGE-Illumina sequencing

To study the relationship between the miRNA and their targets, we performed SAGE-Illumina sequencing to examine the transcriptional profile of the uteri from delayed implantation and activation. The raw data from SAGE-Illumina sequencing is available at Gene Expression Omnibus (GEO: GSE19473). There were 9,912,459 and 12,869,487 reads sequenced from delayed implantation and activation, respectively. After removing the reads with 0/1 or 1/0 in both libraries, there were 8,683,980 and 11,039,265 meaningful reads remained for delayed implantation and activation, respectively. Of the meaningful reads, 356,981 and 348,124 reads were mapped to unigenes for delayed implantation and activation, respectively (Table 8). Among the total unique reads, 51,727 and 51,766 unique reads were identified for delayed implantation and activation, respectively. Of the unique reads, 45,147 (87.28%) and 45,144 (87.21%) unique reads were mapped to one gene for delayed implantation and activation, respectively. There were 10.54% tags for delayed implantation and 10.55% for activation matched to two genes. The remaining 2.18% for delayed implantation and 2.24% for activation matched to multiple genes (Table 8).

**Table 8:** Unique reads of uterine mRNAs.

Category	Delay	Delay (%)	Activation	Activation (%)
Total tags	9,912,459		12,869,487	
Total tags without single tag (0/1 or 1/0)	8,683,980		11,039,265	
Unique tags	356,981	-	348,124	-
Unique tags without single tag (0/1 or 1/0)	151,876	-	155,218	-
Unique tags mapping to unigene	51,727	100	51,766	100
Unique tags mapping to unigene(single match)	45,147	87.28	45,144	87.21
Unique tags mapping to unigene(two match)	5,451	10.54	5,463	10.55
Unique tags mapping to unigene(multiple match)	1,129	2.18	1,159	2.24

In both libraries, the top 30 most abundant tags were mainly matched to genomic repeat sequences and ribosome-related proteins (Table S2). A high percentage of the top 30 abundant tags were shared in both libraries. The counts from the two libraries (delayed and activated uterus) for each gene were compared by z-test and Bonferroni multiple test correction. Genes were designated to be significantly differentially expressed if the *p* value was <0.001, and there was at least a 1.5-fold change in sequence counts between the two libraries. Under these standards, there were 3,033 genes up-regulated and 1,417 genes down-regulated during activation. However, based on the standards that TPM was >100 in either library and the ratio of activation over delayed implantation was ≥2, there were 268 genes up-regulated and 295 genes down-regulated (Table 9, full list in Table S3).

**Table 9:** The top 20 Up-regulated and down-regulated genes among differentially expressed genes in mouse uterus during activation compared to delayed implantation from SAGE-Illumina sequencing.

Tag sequence	Activation(TPM)	Delay (TPM)	Folds	Gene symbol	GO category
CCACTTCCCACAAAAT	6	103	−16.67	Sox17	angiogenesis
CACCGGCCCTGGCACC	18	211	−12.5	Cldn3	cell adhesion
TCCCTGAGTTCGAGGC	15	148	−10	Cdh1	cell adhesion
TTAGAGAAGGAGACAG	143	8	18.55	Birc5	cell cycle
CCTGATGCAAGCTGGC	246	21	11.62	Ube2c	cell cycle
CTTGTAGATATTCACG	39	379	−10	Osr2	cell cycle
ATTAAAACCTTCAAGC	4,160	320	12.98	Actg2	cytoskeleton
CCTTGGGGCCCGATGA	279	24	11.82	Timp1	extracellular region
GTTCAGAGTGGACTGA	203	4	53.3	Sct	hormone activity
AGGAGGGTCAGCTGTG	9	111	−12.5	Ces3	metabolic process
GGGAAGTACGCAAAAT	212	18	11.85	Ass1	metabolic process
CTTAGCAAGGCAATGT	329	0	951.58	Guca2b	metabolic process
AGTTTCCTTGATTATT	713	8	92.42	Rrm2	metabolic process
CATGACATCCGCTGGA	1,456	118	12.28	Gpx3	metabolic process
TCTGACAGAGCCCATT	13	219	−16.67	4833423E24Rik	metabolic process
CTGCAGGCCCTGGGTG	33	399	−12.5	Gstm1	metabolic process
ATTGTCACTGACTACA	7	117	−16.67	Inmt	metabolic process
CGCATGGCCTGTGAGG	13	200	−16.67	Aox3	metabolic process
CTACATCCATTCGGCT	14	199	−14.29	Cyp27a1	metabolic process
GTGTTGTTTACCGTTG	288	13	21.59	Cdc2a	metabolic process
CATCAACACATCCAGT	2,033	11	187.77	Prss28	metabolic process
ACGCAGCAGATGCAGA	1,176	6	185.69	Prss29	metabolic process
TTGCATATCATGATGG	276	5	52.04	Tdo2	metabolic process
AGCCGCTCAAGATTCT	109	0	949.48	Psma7	metabolic process
CATTTTTCCCTCTCTG	160	4	39.71	Ccl2	metabolic process
GGGTTCTCAGCGAGGA	314	7	47.87	Ptx3	metabolic process
AAACGTGGCTGAGCGC	358	25	14.44	Cebpb	transcription
TGGTTCCAGAACCGTC	18	324	−16.67	Msx1	transcription
CCCATGACACAGATGA	183	0	397.26	Dio3	unkown
ACAGAGATGATGAAAA	114	6	18.4	2810417H13Rik	unkown
TTGACAGCATAGACCA	131	9	15.11	Prap1	unkown
AGTGACATGTCTACTG	3	373	−100	Calb1	unkown
TTCATGACTCTTGAGT	4	371	−100	9930023K05Rik	unkown
TTGGTCACCTTCCTCT	3	226	−100	AW011956	unkown
AGTAGGAAGCACAGGT	6	288	−50	Thrsp	unkown
TGACCTCCTCTTCTGG	7	116	−16.67	Fam83a	unkown
CCCACCATCTCACCCA	8	125	−14.29	Pdzk1ip1	unkown
TGTCATGCCAACCTAC	12	156	−14.29	OTTMTSG00000002043	unkown
TAAGCACCTTCTCTCT	11	142	−12.5	Gm967	unkown
TCAGGGTTCCCATGGT	11	105	−10	Pik3ip1	unkown

Note: TPM>100, fold>2, p<0.01.

### Integrative analysis of miRNA and mRNA expression data

**Figure 6:**
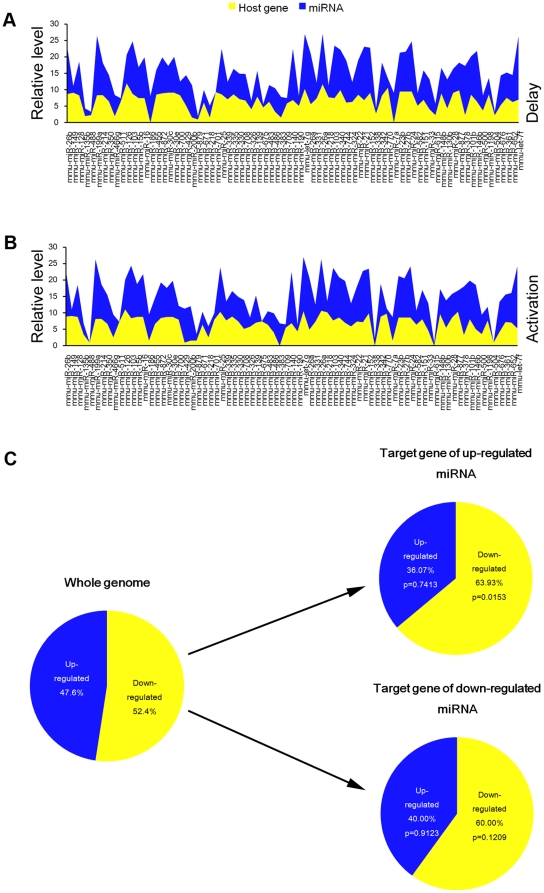
The integrative analysis on miRNA and mRNA data. The relationship between miRNAs and host genes in delayed implantation (A) and activation (B). (C) The enrichment of corresponding target mRNAs of differentially expressed miRNAs. For comprehensive prediction of miRNA target genes, two publically available algorithms (TargetScan and PITA) are used to select the overlapping targets for further analysis.

Because most mammalian miRNAs are intragenic and transcribed as part of their hosting transcription units [20], we hypothesized that the expression profiles of mature miRNAs and their host genes are directly correlated. miRNA expression was compared with their host mRNA expression to see whether they were co-expressed. The list of mouse intragenic miRNAs and corresponding host genes was retrieved from miRBase (Release 13.0, March 2009). Only those genes were considered as host genes if RefSeq sequences were overlapped with the miRNA either in introns, exons or UTR and were transcribed on the same strand as the miRNA. Based on our data from both miRNA Illumina sequencing and mRNA SAGE-Illumina sequencing, we found that miRNA expression was tightly correlated with the host mRNA expression (r = 0.35, p = 0.002 in delayed implantation and r = 0.37, p = 0.001 in activation), suggesting that both miRNA and the host mRNA were co-transcribed (Fig. 6A, B).

In this study, 9 miRNAs were up-regulated and 16 down-regulated in the activated uterus compared to delayed implantation (fold>1.5, TPM>100 and p<0.001). There were 268 up-regulated genes and 295 down-regulated genes in the activated uterus compared to delayed implantation (fold>2, TPM>100 and p<0.001). For comprehensive prediction of miRNA target genes, the results from two publically available algorithms (TargetScan and PITA) were merged. In total, 53 genes (with repeats, 49 unique genes) were predicted to be the targets of up-regulated miRNAs in activated uterus, while 196 (with repeats, 110 unique genes) genes were predicted as targets of down-regulated miRNAs. In our gene expression data, there were 52.4% down-regulated genes and 47.6% up-regulated genes. Among the target genes of the up-regulated miRNAs predicted by either TargetScan or PITA, 63.93% of the target genes predicted was really down-regulated in our study, suggesting the target genes of up-regulated miRNAs during activation were significantly enriched. However, 40.0% of the target genes of down-regulated miRNAs were up-regulated in our study, which is consistent to our gene expression data (47.6%), suggesting that the target genes of down-regulated miRNAs were not enriched (Fig. 6C).

We defined a coherent target of a miRNA as a predicted target if its expression had a reverse pattern with the miRNA. Among the predicted targets of up-regulated miRNAs, down-regulated genes detected by SAGE-Illumina sequencing are considered as coherent targets and otherwise as non-coherent targets. For the down-regulated miRNA targets, up-regulated genes are coherent and otherwise non-coherent. Therefore, 44 genes (with repeat, 39 unique genes) were coherent target of up-regulated miRNAs in activated uterus. 76 genes (with repeats, 44 unique genes) were coherent target of down-regulated miRNAs. The differentially expressed miRNAs and their coherent mRNA targets were listed in Table S4.

**Figure 7:**
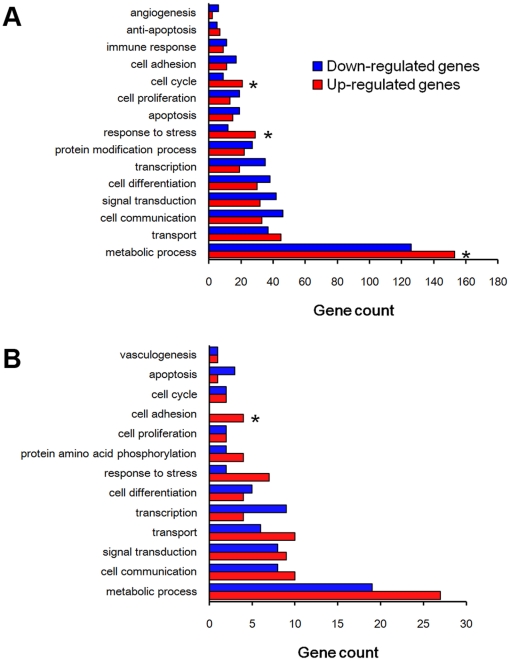
Gene Ontology analysis. (A) The differentially expressed genes from SAGE-Illumina sequencing data. (B) The coherent target genes of the differentially expressed miRNAs. Gene ontology analysis is performed with DAVID tools (http://david.abcc.ncifcrf.gov/tools.jsp). The enrichment p-values are corrected by Benjamini's methods.

Based on Gene Ontology analysis for the differentially expressed genes, the genes involved in cell cycle, response to stress, and metabolic process were significantly enriched in activation group compared to delayed implantation (Fig. 7A). When considering the coherent target genes of the down-regulated miRNAs, the genes involved in cell adhesion were significantly enriched among the up-regulated genes (Fig. 7B).

## Discussion

### General comparison with other deep sequencing data

In our study, the percentage of miRNAs is 74.52% in delayed and 76.57% in activated uteri, respectively. The top 3 most abundant miRNAs are let-7c, let-7f and let-7a. These data are similar to that in mouse oocytes [21]. In amphioxus, the length distribution peaked at 22 nt and almost half of these clean reads (45.11%) are 22 nt in length [22]. In our study, 22 nt is also the dominant size among the small RNAs, consistent with the common size of miRNAs. Additionally, we found that the most abundant sequence of miRNA-143 is not the reference sequence reported. Kuchenbauer et al. [9] also found many isoforms for miR-181a and that the miRBase reference sequence was not the dominant species. However, qRT-PCR widely used for miRNA quantification is mainly based on reference sequence [19]. Therefore, the amount of the reference sequence from qRT-PCR may not reflect the amount of the dominant form of each miRNA. In our previous study, miR-21 at implantation site is up-regulated compared to inter-implantation sites based on Northern blot [8]. However, by using qRT-PCR, miR-21 at implantation is down-regulated compared to inter-implantation site in this study. Similar situation happens for miR-21 during delayed implantation and activation. Our data suggest that it should be cautious when miRNA expression level is examined by different methods. Since data from in situ hybridization and Northern blot are from the hybridization between probes and matched sequences, their data should be more closely related. The data from qRT-PCR should be matched with the reference sequence in Illumina sequencing data.

### Editing and possible significance of mature miRNAs

In our study, 41.5% of let-7a and 64.4% of miR-143 sequences are either edited or alternatively spliced. Among 26 cell types from human, mouse and rat, there are approximately 20% of miRNA mismatches compared with their genomic sequences, including A to I editing (identified as A to G editing), and 3′ terminal A and U additions [23]. Through massively parallel sequencing, ∼50% of the mature miRNAs in the let-7 family display internal insertion/deletions and substitutions when compared to precursor miRNA and the mouse genome reference sequences [10]. For let-7a in mouse ovary, there are 35% exhibited terminal nucleotide additions and/or excisions among the sequences that aligned with pre-miR precursors [10]. A to I editing is the dominant one in several reports [10], [11]. Although A to I editing was indeed found in our data, but not the dominant one. T to A editing is the most dominant form among all of the miRNAs in our study. It is shown that A to I editing in mice is often lower than that in human [23]. It is possible that the mechanism of miRNA editing may be different among mammals. The mechanism on high percentage of T to A editing is still unknown.

IsomiRs resulting from variations at the 5′-end may be of particular interest as they have different seed sequences from the reference miRNA. A to I editing sites also occur within the seed region of mature miRNA sequences, showing that RNA editing can impact miRNA target recognition and function [11], [24]. In our study, a significantly higher percentage of editing occurs within 5′ seed region in delayed implantation group in let-7a, miR-143 and miR-21 compared to activation group. Based on our data, the target genes of unedited miRNAs are largely different from that of edited ones. Once let-7a is edited as let-7a-5C, different sets of target genes are predicted and three target genes are confirmed by luciferase analysis in our study, suggesting that a single G to C base change is sufficient to redirect silencing miRNAs to a new set of targets. For let-7a, miR-143 and miR-21, the editing within seed region in delayed implantation is significantly higher than activation group. However, the significance of this editing is still not clear. A fuller description of the expression of isomiRs for each miRNA would be of interest to determine if there are tissue-specific isomiR distributions involved in development and diseases. RNA editing may contribute to miRNA diversity by generating multiple different miRNAs from an identical miRNA transcript. miRNA editing may simultaneously alleviate and augment the gene-regulation effects of miRNAs by changing the concentration of individual miRNAs [24]. For the physiological significance, let-7a-5C is highly expressed during delayed implantation compared to activation group. Once let-7a is edited into let-7a-5C, there are more genes involved in nucleic acid metabolic process, channel activity, nucleus, biosynthetic process and transcription regulator activity, suggesting that these functions should be suppressed during delayed implantation. This is consistent with low metabolic activity during delayed implantation [4].

*Smad7* was verified as a target gene of let-7a-5C in our study. Both *Smad6* and *Smad7* prevent ligand-induced activation of signal-transducing Smad proteins in the transforming growth factor-β family. In cardiac myofibroblasts, ectopic Smad7 protein is associated with accelerated activation of pro-MMP-2 into MMP-2 [25]. Proper extracellular matrix degradation and blastocyst invasion are essential for embryo implantation and decidualization [26], [27]. MMP-2 is a matrix metalloproteinase and strongly expressed in the stromal cells of mouse uterus during implantation period [27], [28]. In our study, *Smad7* expression is mainly localized in the subluminal stromal cells at implantation sites under activation, suggesting that *Smad7* and MMP-2 should be co-localized at subluminal stromal cells at implantation sites. Therefore, the edited let-7a-5C may play a role for mediating a proper balance between MMP-2 level and embryo invasion for the successful pregnancy through *Smad7*.

### Comparison with implantation-related miRNAs

In our study, there are 20 up-regulated miRNAs and 42 down-regulated miRNAs at least 1.2 folds in activation. Up to date, there is no miRNA expression profile available for comparison with our data from delayed implantation. The closest data for comparison is from our previous paper on miRNA expression from implantation and inter-implantation sites in mouse uterus [8]. Compared to inter-implantation sites, there are 13 miRNAs up-regulated at least 2 folds at implantation sites. Of which, let-7f, let-7d, let-7e, let-7i, miR-20a, miR-298, let-7g, miR-21, and let-7a are up-regulated in activation group. However, miR-26a, let-7b and let-7c and miR-143 up-regulated at implantation sites either doesn't change or is down-regulated in activation group. The reason of this discrepancy is not clear. This may reflect the difference between implantation sites during early pregnancy and activation after delayed implantation. Additionally, miRNAs detected by microarray are based on reference sequences, however, the reference sequences are often not the dominant ones for some miRNAs, which might be another reason causing that discrepancy.

### Comparison with implantation-related mRNA

Ultra-high-throughput sequencing is emerging as an attractive alternative to microarrays for genotyping, analysis of methylation patterns, and identification of transcription factor binding sites [29]. In 10 bp SAGE, around 53,000 tags are obtained from each library [6]. In this study, there are 356,981 tags for delayed implantation and 348,124 tags for activation. The number of tags detected is greatly increased in this study. Additionally, about 50% of the tags are single-matched to genes for 10 bp SAGE [6], while about 87% of tags are single-matched to genes in our 16 bp SAGE. Owing to its increased tag length, long SAGE tags are more reliable in direct assignment to genome sequences. In our data, only about 12% of tags are multiple-matched. Of these tags most tags are mainly B2 repeats and ribosomal proteins.

For delayed implantation, there is only one large scale study on gene expression in mouse uterus using Affymetrix murine expression arrays [5]. They reported 41 down-regulated and 21 up-regulated genes during activation compared to delayed implantation. In our study, there are much more differentially expressed genes detected, including 268 genes up-regulated and 295 genes down-regulated at activation compared with delayed implantation. All of the 21 up-regulated genes in their study are also up-regulated in our study. Among 41 down-regulated genes in their study, 37 genes are also down-regulated in our study. *Ctss* and *Mecp2*, down-regulated in their study, don't change in our study. *Akr1b7* (−4.41 vs 3.15) and *Gzma* (−2.15 vs 14.82), down-regulated in their study, are up-regulated in our study, respectively. We performed qRT-PCR to solve this discrepancy(data not shown). Based on our qRT-PCR data, *Akr1b1* and *Gzma* are indeed up-regulated at activation group compared to delayed implantation. *Mecp2* and *Ctss* are slightly down-regulated at activation group compared to delayed implantation. Additionally, the whole uterus was used in their study [5], but only the implantation sites of mouse uterus following activation were used in our study, which may also reflect the difference between these two studies. This comparison further shows that our data from SAGE-Illumina sequencing is of great value for further understanding the mechanism on delayed implantation.

Because implantation site of day 5 pregnant mouse uterus is very similar to activation after delayed implantation [5], we compared our data on delayed implantation with implantation study on day 5 of pregnancy [6]. Based on our data, the expression pattern of activation group is closely related to that of implantation site (r = 0.39), and the gene expression pattern under delayed implantation is also related to that of inter-implantation sites (r = 0.46). Additionally, many well-known implantation- or decidualization-related genes are identified in our study, including *Prss28* [30], *Des* [31], *Cebpb* [32], *Il1r1* [33], *Ptx3* [34], Ccnd3[35], *Timp1* [36], *Hoxa10* [37], *Il11ra1* [38], *Fst* [39], *Odc1* [40], *Stathmin 1* [41], *Srm* [40], *Gstm2* [42], and *Calb1* [43].

Based on Gene Ontology analysis, the genes involved in metabolic process, response to stress and cell cycle are significantly enriched in the up-regulated genes of activation group compared to down-regulated genes. When considering the targeting genes of differentially expressed miRNAs, the genes involved in metabolic process, response to stress and vasculogenesis are significantly enriched among the differentially expressed genes in both groups. Among the up-regulated genes, the genes involved in cell adhesion are significantly enriched. Cell adhesion is essential for embryo implantation [44]. When examining the differentially expressed gene profile of blastocysts between dormant and activated blastocysts, the major functional categories of altered genes include the cell cycle, cell signaling, and energy metabolic pathways [4]. During delayed implantation, the uterus remains quiescent and the blastocysts become dormant [45]. Further study on these differentially expressed genes in mouse uterus should be beneficial for better understanding the mechanism on delayed implantation.

Estrogen is essential for on-time uterine receptivity and blastocyst activation in mice [3]. Ovariectomy in the morning of day 4 will lead to blastocyst dormancy. In the model of delayed implantation, estrogen is used to activate the dormant blastocyst and initiate embryo implantation [6], [46]. Therefore, we compared the up-regulated genes in our study with the estrogen-stimulated genes. In mouse uterus, 102 genes are up-regulated at least 2 folds after estrogen treatment for 24 h [47]. In our study, 268 genes are up-regulated by least 2 folds in activation group compared to delayed implantation. Among 268 up-regulated genes, 16 genes are also shown to be differentially regulated in mouse uterus after estrogen treatment [47]. This suggests that at least a part of the up-regulated genes in activation group is regulated by estrogen rather than embryos.

### The integrative analysis of mRNA and miRNAs

In our study, we compared miRNA expression with their corresponding host genes. The expression trend between miRNAs and their host genes is highly correlated for both delayed implantation and activation, suggesting that host genes and miRNAs are co-transcribed. Most known miRNA genes have the same type of promoters as protein-coding genes have [48]. Perfect correlations are also found between the expression profiles of the intronic miRNAs and their host genes [49]. The strong correlation between miRNAs and their host genes indicated that they are derived from the same precursor genes and might be under the control of the same promoter. Therefore, it is possible to predict the expression of their embedded miRNAs through large scale analysis of the miRNA host genes.

Right now the information on large scale proteomic analysis of mouse uterus during embryo implantation is still lacking. However, it is shown that miRNAs could down-regulate some of their targets not only at the translational but also at the transcriptional level [13]. Therefore, it is possible to use the comparative expression analysis of miRNAs and mRNAs to identify mRNA targets of miRNAs. In our SAGE-Illumina sequencing data, there are 52.4% down-regulated genes and 47.6% up-regulated genes. Among the target genes of the up-regulated miRNAs predicted by either TargetScan or PITA, 63.93% of the target genes predicted is really down-regulated, suggesting that the target genes of significantly up-regulated miRNAs during activation are significantly enriched. In our study, the enrichment level of down-regulated genes is not very high and the up-regulated genes are not significantly enriched, suggesting that some genes might not be regulated at the transcriptional, but at translational level. In MCF7 cells, the target genes of 14 down-regulated miRNAs are significantly enriched, indicating the strong inverse correlation between miRNA and target gene expression might be essential in gene regulation during the acquisition of fulvestrant resistance [50]. In Drosophila, miRNAs and genes encoding predicted miRNA targets are expressed in a largely mutually exclusive manner [51]. Recent evidences suggest the involvement of miRNAs in tuning the expression of target genes to physiologically relevant levels [52]. Therefore, the differentially expressed miRNAs during embryo implantation may be essential through regulating the mRNA level of their corresponding target genes.

In conclusion, many differentially expressed miRNAs and mRNAs were identified in mouse uterus under delayed implantation and activation in our study. For miRNAs, there are 20 miRNAs up-regulated and 42 miRNAs down-regulated at least 1.2 folds under activation compared to delayed implantation. For mRNAs, there are 268 genes up-regulated and 295 genes down-regulated at least 2 folds under activation compared to delayed implantation. Both miRNA and mRNA expression patterns are closely related and the target genes of up-regulated miRNAs are significantly enriched. There is a higher percentage of miRNA editing at positions 4 and 5 of mature miRNAs under delayed implantation than that under activation. Our data will shed light on further study of mouse embryo implantation.

## Materials and Methods

### Animal treatments

Mature mice (Kunming White outbred strain) were maintained in a controlled environment with a 14-h light/10-h dark cycle. All animal procedures were approved by the Institutional Animal Care and Use Committee of Xiamen University (XMUEA-0080). Female mice were mated with fertile males of the same strain to induce pregnancy (day 1 is the day of vaginal plug). To induce delayed implantation, pregnant mice were ovariectomized under ether anesthesia at 08:30–09:00 h on day 4 of pregnancy. Delayed implantation was maintained from days 5–7 by injecting progesterone (1 mg/mouse, Sigma) in the morning. Estradiol-17β (25 ng/mouse, Sigma) was given to progesterone-primed delayed implantation mice to initiate implantation on day 7 of pregnancy. The mice were sacrificed to collect uteri 24 h after estrogen treatment for activation group. Delayed implantation was confirmed by flushing the blastocysts from one horn of the uterus. The implantation sites of activated uterus were identified through intravenous injection of 0.1 ml of 1% Chicago blue. Uterine tissues were collected from at least 20 mice undergoing delayed implantation and activation, respectively. Equal amounts of uterine tissues from delayed implantation and activation groups were subject to the following Illumina sequencing analysis.

### Illumina sequencing of small RNAs

Total RNAs of delayed and activated uterus were extracted by TRIzol (Invitrogen), followed by a 15% Tris-borate-EDTA urea gel electrophoresis. Small RNAs were separated by the size of 18–30 bases from the gel. After purification, small RNAs were ligated to a 5′ RNA adapter (5′-GUUCAGAGUUCUACAGUCCGACGAUC-3′). Followed by another TBE gel purification and ligated to a 3′ RNA adapter (5′- pUCGUAUGCCGUCUUCUGCUUGidT-3′)(idT is an inverted deoxythymidine), the purified small RNAs were reverse transcribed using Illumina's small RNA RT-Primer (5′-CAAGCAGAAGACGGCATACGA-3′) and amplified by a 15 cycle PCR using Illumina's small RNA primer set (5′-CAAGCAGAAGACGGCATACGA-3′ and 5′-AATGATACGGCGACCACCGA-3′). PCR products were purified and quantified for Illumina sequencing in Shenzhen Huada Gene Sci-Tech Company (Shenzhen, China).

The 5′ end of the read was treated as 5′ nucleotide of the small RNA, and the 3′ end of the small RNA was determined by the 3′ most perfect match to the first 8 nt of the 3′ adaptor. After the reads without a perfect 8-nt adaptor match were deleted, the remaining reads were retrieved from libraries of delayed and activated uteri, respectively. The adaptor-free reads were aligned to mouse genome mm9 (http://hgdownload.cse.ucsc.edu/downloads.html) by Bowtie alignment tool (http://bowtie-bio.sourceforge.net/index.shtml) [53]. The positions of all miRNA genes were also downloaded from miRBase and used for this positional annotation (http://microrna.sanger.ac.uk/sequences/ftp.shtml). Small RNA annotations other than miRNA were downloaded from piRNABank (http://pirnabank.ibab.ac.in/) [54] and fRNAdb (http://www.ncrna.org/frnadb/) [55]. Each of these reads was classified as known miRNA, piRNA, tRNA, rRNA, snRNA, snoRNA, mRNA, genomic sequence or unknown sequences.

The comparison of miRNA copies between our two deep sequencing libraries was performed according to the Z-test algorithm as described previously [56]. For a miRNA, *n*_1_and *n_2_* are the read number of this miRNA and*N*_1_and *N*_2_ are the total number of reads in each library, respectively. The z-statistic is calculated according to the following formula:
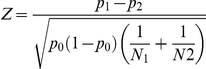
in which 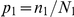, 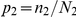 and 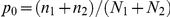.

Two-sided p-value was calculated from z-statistic and followed by Bonferroni multiple test correction. The same method was applied for digital gene expression data in this study.

### Detection of microRNA editing

miRNAs with known SNPs [18] in mature sequences were excluded for further analysis. In order to avoid cross-mapping of small RNA reads, a rough alignment was performed with Bowtie software. The potential read/known microRNA sequence pairs were then aligned by Needleman-Wunsch dynamic programming algorithm. The penalty scores for perfect match, mismatch, gap opening and gap extension were set for 1, −1, −2 and −1, respectively. Only the alignment with the highest score for each read was kept for further analysis.

Statistical analysis was performed as previous described [10]. The test is based on the null hypothesis where all positions behave the same with respect to base modification. The editing rate of each nucleotide position was calculated. Then the editing rate was transformed into standardized score (Z-score). The median Z-score across all positions was set to 0. A p-value was assigned to each Z-score according to normal distribution. A significant editing which is much higher than background noise (sequencing error) was considered if a p value is less or equal to 0.01.

### SAGE-Illumina sequencing analysis of mRNAs

Total RNAs of delayed and activated uteri were extracted by TRIzol. Total RNA quality from both delayed and activation groups was comparable based on analysis with a Bioanalyzer 2100 (Agilent). The mRNA library for sequencing was prepared using Gene Expression Sample Prep Kit (Illumina) according to the manufacturer's instructions. Briefly, mRNAs were isolated through binding to a magnetic oligo(dT) beads. First strand cDNA was synthesized using the binding mRNA as a template, and followed by the synthesis of the second strand of cDNA. After cDNAs were digested with DpnII, the double stranded cDNA fragments attached to the oligo(dT) beads were collected and ligated to a GEX DpnII adapter 1 at the site of DpnII cleavage. The sequence for MmeI was included in GEX DpnII adapter 1. After purification, products coupled with oligo(dT) beads were digested with MmeI to create 16 bp tags, followed by ligating to a GEX adapter 2 at the site of MmeI cleavage. After PCR amplification, the purified DNA fragments were used directly for sequencing using the Illumina Cluster Station in Shenzhen Huada Gene Sci-Tech Company.

After sequencing, 16 bp tags were extracted from SAGE-Illumina sequencing libraries by in-house perl scripts. Then the tags were mapped to Unigene build 21. SAGEmap algorithm was used for tag-to-gene mapping with slightly modifications. Briefly, Unigene build 21 data were downloaded from NCBI. For each sequence in the Unigene database, 16 base tags adjacent to the 3′-most anchor enzyme DpnII site (GATC) were extracted.

The sequences were divided into 4 types: (a) mRNA: tags from GenBank submission transcripts which have poly(A) tails and/or signals; (b) High-throughput sequencing: tags from high throughput sequencing transcripts which have either poly(A) tails and/or signals; (c) ESTs with poly(A) tails: tags from EST sequences which have poly(A) tails and/or signals in the same orientation as the tags. (d) Sequences without poly(A) tails: all sequences of which no polyadenylation signal or tail was found. For each tag-UniGene pair, a reliable score was calculated as:
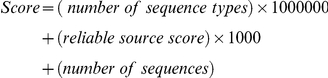



The number of sequence types could range from 1 to 4 depending on whether the tag-UniGene pair was defined as mRNA, mRNA sequences from high-throughput sequencing, ESTs with poly(A) tails, and/or sequences without poly(A) tails. The reliable source score  =  (number of mRNA + number of mRNA sequences from high-throughput sequencing) +0.5× number of ESTs with poly (A) tails. The higher the score of a tag-UniGene pair, the more reliable the mapping is considered. When more than two UniGene clusters were assigned to a certain tag, only the two UniGene clusters with the highest scores were chosen.

### qRT-PCR analysis for mature miRNAs, precursor miRNAs and mRNAs

Total RNAs were extracted from delayed or activation mouse uteri with TRIzol reagent, digested with DNase I and reverse-transcribed into cDNA with PrimeScript™ RT reagent Kit (TaKara, Dalian, China). For mature miRNAs, there were 0.1 µg total RNAs, 2 µl PrimeScript Buffer, 25 nM stem-loop RT primers, and 0.5 µl PrimeScript RT Enzyme Mix I in 10 µl volume. Reverse transcription was performed at 16°C for 30 min followed by 60 cycles of 30°C for 30 sec, 42°C for 30 sec and 50°C for 1 sec, and ended at 85°C for 5 min. qRT-PCR was performed using a SYBR Premix Ex TaqTM kit on the Rotor-Gene 3000A system at 95°C for 10 sec, followed by 40 cycles of 95°C for 5 sec, 60°C for 5 sec and 72°C for 8 sec. For precursor miRNAs, 50 nM special reverse primers were used as RT primers. For mRNA quantification, 2.5 µM Oligo dT Primer and 5.0 µM Random 6 mers were used for reverse transcription and qRT-PCR was performed at 95°C for 10 sec, followed by 40 cycles of 95°C for 5 sec and 60°C for 34 sec. Primers used for qRT-PCR were listed in Table S5. Mouse rPL7 gene was amplified as a reference gene for normalization.

### Dual-luciferase activity assay

The 3′-UTR segment of each mouse gene predicted as target genes of let-7a-5C (C at the 5th position of let-7a) or let-7a (the reference sequence of let-7a, G at the 5th position) was amplified by PCR from mouse cDNAs and inserted into the downstream of luciferase reporter gene in the pGL3 control vector. Primers used for amplifying 3′-UTR of each mouse gene were listed in Table S5. The plasmid pRL-TK containing renilla luciferase was co-transfected for data normalization. Transfection and dual luciferase analysis was performed as described previously [8].

### Primary culture of uterine stromal cells

Uterine stromal cells were cultured as described previously [8]. Uterine horns from mice on day 4 of pregnancy were cleaned of fat tissues, slit longitudinally, and washed thoroughly in Hanks' balanced salt solution (HBSS, Sigma) without Ca^2+^/Mg^2+^ and phenol red. Tissues were then placed in 5 ml of fresh medium (HBSS with antibiotics) containing 6 mg/ml dispase (Gibco-BRL) and 10 mg/ml trypsin (Sigma), and incubated for 1 h at 4°C, 1 h at room temperature, and then 10 min at 37°C, respectively. Following the digestion, tissues were shaken several times to dislodge the sheet of luminal epithelial cells. The remaining tissues were washed three times in fresh medium and digested in HBSS containing 0.15 mg/ml collagenase (Gibco-BRL) at 37°C for 30 min. Following digestion and shaking, the digested cells were passed through a 70-µm filter to get rid of residual epithelial sheets and centrifuged. The cells were plated in 24-well culture plates and cultured in DMEM containing 10% FBS.

### Western blot

Cultured cells or uterine tissues were collected in lysis buffer (50 mM Tris-HCl, pH 7.5, 150 mM NaCl, 1% Triton X-100, and 0.25% sodium deoxycholate) and briefly sonicated to shear DNA and reduce sample viscosity. Protein concentration was measured by BCA Reagent kit (Applygen, Beijing, China). Samples were run on a 10% PAGE gel and transferred onto nitrocellulose membranes. After blocked in 5% nonfat dry milk in TPBS (0.1% Tween 20 in PBS) for 1 h, membranes were incubated with monoclonal anti-human SMAD7 Antibody (R & D Systems) overnight at 4°C. After three washes in 5% milk/TPBS 10 min each, membranes were incubated in goat anti-mouse IgG conjugated with horseradish perioxidase for 1 h followed by two washes in 5% nonfat milk in TPBS, TPBS and PBS 5 min each, respectively. The signals were developed in ECL Chemiluminescent kit (Amersham Pharmacia Biotech, Arlington Heights, IL).

### In situ hybridization

In situ hybridization was performed as we previously described [6]. Briefly, frozen uterine sections were hybridized with the digoxigenin-labeled Smad7 RNA probes. All of the sections were counterstained with 1% methyl green.

## Supporting Information

Table S1 **The 30 most abundent miRNAs in delayed and activated uterus.**
(DOC)

Table S2 **The top 30 most abundant tags in mouse uterus from digital gene expression.**
(DOC)

Table S3 **Full list of differentially expressed genes from Digital gene expression data.**
(DOC)

Table S4 **The differentially expressed miRNAs and their corresponding coherent target mRNAs during delayed implantation.**
(DOC)

Table S5 **Primers used in this study.**
(DOC)
