# Supplementary material for: The Integrative Analysis of microRNA and mRNA Expression in Mouse Uterus under Delayed Implantation and Activation
Source: PLoS One. 2010 Nov 29;5(11):e15513. doi: 10.1371/journal.pone.0015513 (PMC2993968; doi:10.1371/journal.pone.0015513)
Supplement: Table S1 — The 30 most abundent miRNAs in delayed and activated uterus. (DOC) [file pone.0015513.s001.doc]

Table S1 The 30 most abundent miRNAs in delayed and activated uterus

|  | TPM | | Folds |  |
| --- | --- | --- | --- | --- |
| miRNA name | Activation | Delay | (Activation/Delay) | p value |
| mu-let-7c | 317,116 | 336,169 | -1.06 | 0 |
| mmu-let-7f | 128,615 | 101,036 | 1.27 | 0 |
| mmu-let-7a | 114,069 | 95,504 | 1.19 | 0 |
| mmu-let-7b | 94,695 | 113,963 | -1.20 | 0 |
| mmu-miR-199b | 56,483 | 65,100 | -1.15 | 0 |
| mmu-miR-1 | 26,749 | 22,461 | 1.19 | 0 |
| mmu-miR-29a | 18,049 | 26,525 | -1.47 | 0 |
| mmu-let-7d | 24,228 | 18,731 | 1.29 | 0 |
| mmu-let-7g | 20,308 | 19,536 | 1.04 | 0 |
| mmu-miR-140* | 14,259 | 16,701 | -1.18 | 6.37E-208 |
| mmu-let-7e | 13,722 | 11,554 | 1.19 | 0 |
| mmu-miR-21 | 14,877 | 10,297 | 1.44 | 0 |
| mmu-let-7i | 12,975 | 12,151 | 1.07 | 0 |
| mmu-miR-143 | 12,400 | 12,264 | 1.01 | 1 |
| mmu-miR-320 | 13,741 | 10,754 | 1.28 | 0 |
| mmu-miR-26a | 7,686 | 11,285 | -1.47 | 0 |
| mmu-miR-103 | 8,411 | 9,165 | -1.09 | 3.60E-34 |
| mmu-miR-24 | 7,121 | 10,058 | -1.41 | 0 |
| mmu-miR-378 | 6,561 | 6,211 | 1.06 | 1.58E-09 |
| mmu-miR-191 | 5,577 | 5,613 | -1.01 | 1 |
| mmu-miR-146b | 8,297 | 2,355 | 3.52 | 0 |
| mmu-miR-30a | 4,176 | 5,033 | -1.20 | 4.98E-85 |
| mmu-miR-181a | 3,579 | 4,767 | -1.33 | 5.76E-181 |
| mmu-miR-423-5p | 4,273 | 3,452 | 1.24 | 0 |
| mmu-miR-26b | 3,604 | 4,033 | -1.12 | 3.66E-25 |
| mmu-miR-185 | 3,554 | 3,111 | 1.14 | 0 |
| mmu-miR-107 | 2,469 | 2,757 | -1.11 | 2.62E-16 |
| mmu-miR-152 | 2,653 | 2,551 | 1.04 | 0.40 |
| mmu-miR-25 | 2,514 | 2,175 | 1.16 | 0 |
| mmu-miR-10a | 1,839 | 2,666 | -1.45 | 3.88E-162 |
