# Supplementary material for: The Integrative Analysis of microRNA and mRNA Expression in Mouse Uterus under Delayed Implantation and Activation
Source: PLoS One. 2010 Nov 29;5(11):e15513. doi: 10.1371/journal.pone.0015513 (PMC2993968; doi:10.1371/journal.pone.0015513)
Supplement: Table S2 — The top 30 most abundant tags in mouse uterus from digital gene expression. (DOC) [file pone.0015513.s002.doc]

Table S2 The top 30 most abundant tags in mouse uterus from digital gene expression

| Tag name | Activation | Delay | Symbol | Description |
| --- | --- | --- | --- | --- |
| TGACTCCCTCTTCTGG | 453,550 | 102,210 | B2_repeat | Genomic repeat |
| TGATGCCCTCTTCTGG | 304,971 | 67,930 | B2_repeat | Genomic repeat |
| GCCTGGAGAAATGACC | 127,160 | 84,559 | Rpl41 | Ribosomal protein L41 |
| TGACGCCCTCTTCTGG | 118,380 | 39,938 | B2_repeat | Genomic repeat |
| GCATTGCCAAGGAGGA | 67,208 | 63,091 | Rpl3 | Ribosomal protein L3 |
| TTTATAATTTGAGAGG | 41,819 | 82,826 | Cox1 | Cytochrome c oxidase subunit I |
| TGCGGTGACTTGATGT | 52,009 | 62,136 | Rps24 | Ribosomal protein S24 |
| TCTCTTCCCAGAGGAA | 36,208 | 66,798 | Rps4x | Ribosomal protein S4, X-linked |
| TGCTTCAATAATTTAA | 38,399 | 58,629 | Cox2 | Cytochrome c oxidase subunit II |
| CGTGTGAAACTGGATG | 44,467 | 52,324 | Rps7 | Ribosomal protein S7 |
| ATAAAATTTGACAATG | 71,835 | 24,430 | Rps23 | Ribosomal protein S23 |
| TGAAATGAACCAGCCC | 49,501 | 42,564 | Rpl38 | Ribosomal protein L38 |
| GCTGGACAGGTGGCAG | 48,166 | 40,984 | Rps19 | Ribosomal protein S19 |
| AAACAACCCAACAGGA | 36,312 | 52,619 | Cytb | Cytochrome b |
| GGGGTTTACCTTTGTA | 46,320 | 37,700 | Rps16 | Ribosomal protein S16 |
| ATCTAAACTGAGTCCA | 47,166 | 33,167 | Rpl23a | Ribosomal protein L23a |
| TCACAGGCTGAGAAAT | 56,432 | 23,602 | Sparc | Secreted acidic cysteine rich glycoprotein |
| CATCTGCATTTGCGGC | 39,378 | 38,642 | Rplp0 | Ribosomal protein, large, P0 |
| AATGCCCTCATTAAAG | 41,766 | 35,032 | Rplp1 | Ribosomal protein, large, P1 |
| GAGAAGATGCAGGAGT | 40,411 | 35,240 | Rpl26 | Ribosomal protein L26 |
| TGTGCAGGGTATTAAC | 42,244 | 31,317 | Actg1 | Actin, gamma, cytoplasmic 1 |
| AACAAATCTCCTAGGC | 43,540 | 27,601 | ZXDB | Similar to Zinc finger X-linked protein ZXDB |
| GGAAGATGGTGGGTGA | 44,601 | 25,505 | Ifitm3 | Interferon induced transmembrane protein 3 |
| TATCACCTGTCACCAT | 26,783 | 42,061 | Tpt1 | Tumor protein, translationally-controlled 1 |
| ACAGAGCTGGGAAGAT | 28,910 | 39,462 | Rps15a | Ribosomal protein S15A |
| CACAAGCGACTCATTG | 26,644 | 40,467 | Rps20 | Ribosomal protein S20 |
| GGTGCCACCCACTCCT | 37,422 | 29,059 | Rps15 | Ribosomal protein S15 |
| ATATGCCAAACCCTCT | 27,795 | 38,193 | B2m | Beta-2 microglobulin |
| CGGAAGAAGATGATGG | 26,510 | 35,463 | Rps3a | Ribosomal protein S3A |
| TGAAGGTGGAATTGTC | 36,204 | 25,285 | Rpl35 | Ribosomal protein L35 |
