# Supplementary material for: The Integrative Analysis of microRNA and mRNA Expression in Mouse Uterus under Delayed Implantation and Activation
Source: PLoS One. 2010 Nov 29;5(11):e15513. doi: 10.1371/journal.pone.0015513 (PMC2993968; doi:10.1371/journal.pone.0015513)
Supplement: Table S3 — Full list of differentially expressed genes from Digital gene expression data. (DOC) [file pone.0015513.s003.doc]

Table S3 Full list of differentially expressed genes from Digital gene expression data

| GO category | Activation | Delay | Folds | p value | Gene Symbol |
| --- | --- | --- | --- | --- | --- |
| angiogenesis | 6 | 103 | -16.67 | 2.50E-231 | Sox17 |
|  | 22 | 124 | -5.56 | 4.10E-173 | Fgfr2 |
|  | 59 | 263 | -4.55 | 0 | Anpep |
|  | 196 | 457 | -2.33 | 1.64E-243 | Egfl7 |
|  | 71 | 204 | -2.86 | 2.43E-149 | Igf1 |
|  | 190 | 46 | 4.1 | 3.89E-183 | Cryab |
| antigen processing and presentation | 57 | 164 | -2.86 | 3.79E-121 | H2-Ea |
|  | 117 | 329 | -2.78 | 2.34E-236 | H2-Ab1 |
|  | 75 | 188 | -2.5 | 2.92E-113 | H2-Eb1 |
| apoptosis | 20 | 107 | -5.26 | 7.75E-144 | Ypel3 |
|  | 36 | 111 | -3.13 | 9.39E-90 | Rnf130 |
|  | 43 | 106 | -2.44 | 1.68E-60 | Bnip3l |
|  | 43 | 104 | -2.44 | 1.06E-58 | Tial1 |
|  | 231 | 543 | -2.33 | 3.94E-292 | Gsn |
|  | 216 | 101 | 2.14 | 1.38E-81 | Dad1 |
|  | 419 | 187 | 2.24 | 1.85E-175 | Clptm1l |
|  | 636 | 204 | 3.13 | 0 | Pdia3 |
|  | 144 | 41 | 3.52 | 7.24E-119 | Tmbim6 |
| calcium ion binding | 65 | 357 | -5.56 | 0 | Pls3 |
|  | 46 | 161 | -3.45 | 6.92E-149 | Anxa4 |
|  | 66 | 219 | -3.33 | 3.28E-194 | Clec3b |
|  | 39 | 118 | -3.03 | 4.03E-92 | Sgce |
| cell adhesion | 18 | 211 | -12.5 | 0 | Cldn3 |
|  | 15 | 148 | -10 | 5.19E-279 | Cdh1 |
|  | 25 | 173 | -6.67 | 5.60E-277 | Ezr |
|  | 24 | 103 | -4.35 | 2.33E-117 | Lama2 |
|  | 27 | 103 | -3.85 | 6.36E-105 | Lama3 |
|  | 222 | 814 | -3.7 | 0 | Tgfbi |
|  | 121 | 428 | -3.57 | 0 | Dpt |
|  | 50 | 176 | -3.57 | 1.59E-166 | Clstn1 |
|  | 47 | 120 | -2.56 | 2.33E-73 | Pecam1 |
|  | 75 | 170 | -2.27 | 3.61E-84 | Cdh5 |
|  | 296 | 614 | -2.08 | 1.97E-259 | Pcp4 |
|  | 227 | 103 | 2.2 | 4.06E-91 | Itga5 |
|  | 204 | 70 | 2.93 | 1.63E-134 | Fbln2 |
|  | 136 | 43 | 3.18 | 6.82E-99 | Emilin2 |
|  | 178 | 43 | 4.09 | 1.70E-171 | Itgb5 |
|  | 128 | 27 | 4.67 | 2.26E-138 | Aatf |
|  | 695 | 173 | 4.03 | 0 | Ppib |
| cell cycle | 39 | 379 | -10 | 0 | Osr2 |
|  | 20 | 108 | -5.26 | 1.66E-146 | Sesn1 |
|  | 49 | 111 | -2.27 | 1.07E-54 | Ccnl1 |
|  | 55 | 120 | -2.17 | 1.24E-55 | Spin1 |
|  | 105 | 229 | -2.17 | 1.80E-106 | Ccnl2 |
|  | 158 | 76 | 2.08 | 2.95E-55 | Ilk |
|  | 617 | 294 | 2.1 | 2.11E-224 | Calm1 |
|  | 290 | 122 | 2.37 | 8.28E-136 | Ccnd3 |
|  | 127 | 52 | 2.43 | 2.64E-61 | 7-Sep |
|  | 2418 | 702 | 3.44 | 0 | S100a6 |
|  | 304 | 52 | 5.8 | 0 | Cdk4 |
|  | 202 | 24 | 8.34 | 0 | Cks1b |
|  | 506 | 48 | 10.55 | 0 | H2afx |
|  | 246 | 21 | 11.62 | 0 | Ube2c |
|  | 143 | 8 | 18.55 | 2.77E-291 | Birc5 |
|  | 148 | 52 | 2.82 | 1.09E-91 | Stmn1 |
|  | 57 | 117 | -2.04 | 1.95E-47 | Nck2 |
|  | 34 | 108 | -3.23 | 9.92E-90 | Ttc8 |
|  | 79 | 384 | -4.76 | 0 | Sfi1 |
|  | 33 | 243 | -7.14 | 0 | Smarca2 |
|  | 126 | 279 | -2.22 | 2.99E-135 | Grn |
|  | 190 | 66 | 2.88 | 1.13E-121 | Pcna |
|  | 713 | 8 | 92.42 | 0 | Rrm2 |
|  | 86 | 196 | -2.27 | 5.50E-98 | Dctn2 |
|  | 122 | 49 | 2.49 | 2.06E-61 | Mapk1ip1l |
| collagen | 68 | 256 | -3.7 | 4.91E-258 | Col4a5 |
|  | 656 | 311 | 2.11 | 5.12E-242 | Col4a1 |
|  | 637 | 241 | 2.64 | 0 | Col6a3 |
|  | 139 | 41 | 3.42 | 1.60E-110 | Col14a1 |
|  | 1973 | 403 | 4.9 | 0 | Col3a1 |
| cytochrome | 80 | 161 | -2 | 1.61E-61 | Cyb5b |
|  | 382 | 179 | 2.14 | 2.23E-145 | Cyb5 |
|  | 1396 | 621 | 2.25 | 0 | Cox7c |
|  | 187 | 47 | 3.97 | 3.05E-175 | Cyp26a1 |
|  | 698 | 150 | 4.66 | 0 | Cox7a2 |
|  | 117 | 360 | -3.13 | 1.71E-292 | Cox7a2l |
| cytoskeleton | 23 | 218 | -9.09 | 0 | Krt13 |
|  | 58 | 314 | -5.56 | 0 | Tppp3 |
|  | 42 | 173 | -4.17 | 5.24E-192 | Krt8 |
|  | 41 | 128 | -3.13 | 1.94E-105 | Dync1li2 |
|  | 112 | 287 | -2.56 | 4.83E-178 | Krt18 |
|  | 130 | 304 | -2.33 | 6.16E-161 | Antxr1 |
|  | 86 | 172 | -2 | 7.14E-66 | Mtap4 |
|  | 1406 | 690 | 2.04 | 0 | Tmsb4x |
|  | 1380 | 607 | 2.27 | 0 | Tubb5 |
|  | 902 | 375 | 2.4 | 0 | Tagln |
|  | 1118 | 434 | 2.57 | 0 | Tuba1a |
|  | 172 | 66 | 2.6 | 3.05E-94 | Tubb6 |
|  | 691 | 264 | 2.62 | 0 | Des |
|  | 2296 | 778 | 2.95 | 0 | Csrp1 |
|  | 384 | 125 | 3.07 | 2.15E-270 | Pfn1 |
|  | 176 | 35 | 4.97 | 6.70E-200 | Arpc2 |
|  | 208 | 37 | 5.57 | 1.11E-257 | Ckap4 |
|  | 4160 | 320 | 12.98 | 0 | Actg2 |
|  | 123 | 368 | -3.03 | 1.34E-287 | Myl12b |
| DNA methylation | 42 | 119 | -2.86 | 3.17E-86 | Baz2a |
|  | 154 | 62 | 2.47 | 8.28E-77 | Dnmt1 |
| DNA repair | 756 | 298 | 2.54 | 0 | Morf4l2 |
|  | 163 | 63 | 2.61 | 1.56E-89 | Ube2n |
|  | 157 | 53 | 2.98 | 1.81E-105 | Slk |
|  | 219 | 70 | 3.15 | 9.03E-159 | Sfpq |
|  | 242 | 39 | 6.24 | 0 | Hspa1a |
|  | 315 | 41 | 7.72 | 0 | Hmgb2 |
|  | 109 | 14 | 7.87 | 6.34E-164 | Uhrf1 |
| dosage compensation | 262 | 556 | -2.13 | 7.24E-247 | Xist |
| histone | 53 | 141 | -2.63 | 4.42E-92 | H2afv |
|  | 82 | 212 | -2.56 | 7.27E-134 | H1f0 |
|  | 1580 | 598 | 2.64 | 0 | H2afz |
| metabolic process | 154 | 321 | -2.08 | 9.14E-137 | Agrn |
|  | 130 | 24 | 5.36 | 1.38E-155 | Ext2 |
|  | 35 | 107 | -3.03 | 2.19E-84 | Mprip |
|  | 278 | 94 | 2.96 | 5.70E-186 | Prdx2 |
|  | 102 | 41 | 2.5 | 1.74E-51 | Gadd45g |
|  | 9 | 111 | -12.5 | 1.61E-228 | Ces3 |
|  | 212 | 18 | 11.85 | 0 | Ass1 |
|  | 65 | 207 | -3.23 | 8.72E-175 | Ddah2 |
|  | 291 | 105 | 2.76 | 1.31E-175 | Atp5c1 |
|  | 139 | 47 | 2.98 | 8.28E-93 | Tmem70 |
|  | 99 | 216 | -2.17 | 5.82E-101 | Anxa5 |
|  | 48 | 126 | -2.63 | 1.78E-81 | Adcy6 |
|  | 11 | 117 | -10 | 5.75E-228 | Dcxr |
|  | 108 | 290 | -2.7 | 3.80E-194 | Wbp11 |
|  | 312 | 106 | 2.94 | 4.98E-207 | Glo1 |
|  | 111 | 36 | 3.07 | 9.04E-77 | Mdh1 |
|  | 1066 | 231 | 4.62 | 0 | Ldha |
|  | 49 | 170 | -3.45 | 4.28E-156 | Npr2 |
|  | 329 | 0 | 951.58 | 0 | Guca2b |
|  | 85 | 230 | -2.7 | 3.68E-154 | Hoxa11 |
|  | 405 | 151 | 2.69 | 3.33E-235 | Banf1 |
|  | 246 | 91 | 2.71 | 1.89E-144 | Top2b |
|  | 432 | 171 | 2.52 | 4.30E-226 | Dtymk |
|  | 143 | 22 | 6.45 | 2.72E-193 | Dut |
|  | 64 | 134 | -2.08 | 5.21E-56 | Trip10 |
|  | 889 | 437 | 2.04 | 6.91E-303 | Psmb4 |
|  | 43 | 120 | -2.78 | 3.65E-83 | Gcnt2 |
|  | 315 | 135 | 2.34 | 4.14E-143 | Psmd2 |
|  | 279 | 24 | 11.82 | 0 | Timp1 |
|  | 126 | 41 | 3.11 | 3.66E-89 | Ggh |
|  | 1456 | 118 | 12.28 | 0 | Gpx3 |
|  | 20 | 187 | -9.09 | 0 | Gdpd3 |
|  | 139 | 62 | 2.24 | 4.67E-57 | Pkm2 |
|  | 944 | 384 | 2.46 | 0 | Eno1 |
|  | 835 | 175 | 4.77 | 0 | Pgam1 |
|  | 1824 | 347 | 5.26 | 0 | Pgk1 |
|  | 538 | 173 | 3.12 | 0 | Bud31 |
|  | 169 | 79 | 2.14 | 3.12E-63 | Hbb-b1 |
|  | 73 | 173 | -2.38 | 2.98E-94 | Rbbp9 |
|  | 2061 | 591 | 3.49 | 0 | Hint1 |
|  | 82 | 276 | -3.33 | 1.88E-248 | Gsto1 |
|  | 49 | 146 | -3.03 | 7.12E-114 | Pla2g16 |
|  | 13 | 219 | -16.67 | 0 | 4833423E24Rik |
|  | 42 | 158 | -3.7 | 1.59E-158 | Hadh |
|  | 119 | 663 | -5.56 | 0 | Apoe |
|  | 26 | 167 | -6.67 | 2.82E-259 | Alox15 |
|  | 45 | 150 | -3.33 | 4.13E-132 | Gstz1 |
|  | 33 | 399 | -12.5 | 0 | Gstm1 |
|  | 48 | 332 | -7.14 | 0 | Gsta4 |
|  | 93 | 327 | -3.45 | 0 | Gstm2 |
|  | 37 | 117 | -3.13 | 2.67E-96 | Cbs |
|  | 61 | 190 | -3.13 | 4.32E-157 | Vat1 |
|  | 58 | 124 | -2.13 | 4.11E-55 | Uckl1 |
|  | 69 | 148 | -2.13 | 2.09E-65 | Acot1 |
|  | 154 | 330 | -2.13 | 9.83E-148 | Spint2 |
|  | 234 | 109 | 2.15 | 7.61E-90 | Got2 |
|  | 114 | 52 | 2.19 | 9.60E-45 | Mogs |
|  | 391 | 149 | 2.62 | 7.40E-218 | Nat5 |
|  | 7 | 117 | -16.67 | 1.03E-265 | Inmt |
|  | 127 | 62 | 2.05 | 1.61E-42 | Psmd14 |
|  | 328 | 110 | 2.97 | 2.27E-220 | Ube2d3 |
|  | 424 | 129 | 3.28 | 0 | Ube2s |
|  | 81 | 200 | -2.44 | 3.28E-116 | Mgea5 |
|  | 40 | 108 | -2.7 | 1.61E-72 | Ndufc2 |
|  | 119 | 39 | 3.07 | 2.82E-82 | Lmna |
|  | 36 | 104 | -2.94 | 1.10E-78 | Rev3l |
|  | 105 | 256 | -2.44 | 1.02E-147 | Hp1bp3 |
|  | 158 | 75 | 2.12 | 5.02E-58 | Penk |
|  | 168 | 38 | 4.4 | 2.18E-173 | Odc1 |
|  | 13 | 200 | -16.67 | 0 | Aox3 |
|  | 14 | 199 | -14.29 | 0 | Cyp27a1 |
|  | 25 | 184 | -7.14 | 2.72E-302 | Aox1 |
|  | 190 | 417 | -2.17 | 6.70E-197 | Fmo1 |
|  | 264 | 121 | 2.18 | 6.33E-104 | Gpx8 |
|  | 106 | 33 | 3.24 | 7.37E-79 | Lox |
|  | 251 | 22 | 11.28 | 0 | Cycs |
|  | 294 | 1375 | -4.76 | 0 | Dcn |
|  | 44 | 193 | -4.35 | 7.24E-227 | Pisd |
|  | 148 | 377 | -2.56 | 9.94E-235 | Ctdsp2 |
|  | 237 | 76 | 3.12 | 9.53E-170 | Pdgfc |
|  | 262 | 74 | 3.55 | 2.78E-219 | Pdap1 |
|  | 151 | 74 | 2.03 | 5.56E-50 | Stt3a |
|  | 258 | 114 | 2.27 | 7.93E-111 | Rpn1 |
|  | 61 | 145 | -2.38 | 8.95E-78 | Abca1 |
|  | 161 | 74 | 2.17 | 4.01E-62 | Rpn2 |
|  | 119 | 398 | -3.33 | 0 | Clk1 |
|  | 40 | 114 | -2.86 | 1.38E-82 | Clk4 |
|  | 60 | 170 | -2.86 | 9.66E-124 | Gsk3b |
|  | 60 | 141 | -2.33 | 1.43E-74 | Nrbp2 |
|  | 56 | 127 | -2.27 | 2.09E-63 | Trpm7 |
|  | 54 | 121 | -2.22 | 5.22E-59 | Csnk1e |
|  | 59 | 123 | -2.08 | 1.53E-51 | Ulk1 |
|  | 75 | 152 | -2.04 | 1.16E-59 | Ppp1r12c |
|  | 105 | 50 | 2.11 | 2.77E-37 | Brd4 |
|  | 315 | 137 | 2.3 | 2.51E-138 | Csnk1a1 |
|  | 117 | 45 | 2.58 | 2.06E-62 | Taok1 |
|  | 232 | 75 | 3.1 | 1.04E-164 | Pim3 |
|  | 288 | 13 | 21.59 | 0 | Cdc2a |
|  | 817 | 258 | 3.17 | 0 | Ddost |
|  | 150 | 48 | 3.1 | 4.94E-106 | Psmc5 |
|  | 155 | 49 | 3.19 | 9.73E-114 | Psmb3 |
|  | 106 | 33 | 3.22 | 2.78E-78 | Psma5 |
|  | 201 | 47 | 4.25 | 9.99E-201 | Pdia4 |
|  | 892 | 205 | 4.36 | 0 | P4hb |
|  | 496 | 111 | 4.47 | 0 | Pdia6 |
|  | 260 | 103 | 2.52 | 9.68E-136 | Hspa9 |
|  | 526 | 169 | 3.12 | 0 | Ahsa1 |
|  | 3435 | 976 | 3.52 | 0 | Hspa8 |
|  | 146 | 34 | 4.31 | 4.45E-147 | Hsp90aa1 |
|  | 276 | 37 | 7.38 | 0 | Fkbp11 |
|  | 546 | 227 | 2.4 | 3.82E-263 | Tgfbr2 |
|  | 48 | 186 | -3.85 | 9.31E-195 | Pcmtd1 |
|  | 35 | 118 | -3.33 | 4.63E-106 | Usp11 |
|  | 60 | 172 | -2.86 | 8.34E-126 | Stk38 |
|  | 41 | 105 | -2.56 | 8.83E-66 | Ntan1 |
|  | 50 | 116 | -2.33 | 2.15E-60 | Mgrn1 |
|  | 58 | 133 | -2.27 | 1.02E-66 | Rnf20 |
|  | 154 | 58 | 2.66 | 8.52E-87 | Sepx1 |
|  | 668 | 149 | 4.47 | 0 | Erp29 |
|  | 32 | 110 | -3.45 | 9.08E-101 | Cpne3 |
|  | 178 | 81 | 2.2 | 3.64E-71 | Tomm40 |
|  | 276 | 113 | 2.45 | 1.40E-137 | Tomm20 |
|  | 279 | 742 | -2.63 | 0 | Tgm2 |
|  | 10 | 103 | -10 | 4.37E-197 | Pamr1 |
|  | 173 | 1477 | -8.33 | 0 | Ctsd |
|  | 48 | 188 | -4 | 3.30E-199 | Cndp2 |
|  | 45 | 107 | -2.38 | 1.36E-58 | Prss23 |
|  | 179 | 47 | 3.78 | 6.71E-160 | Adamts15 |
|  | 307 | 79 | 3.9 | 3.16E-283 | Xpnpep1 |
|  | 1176 | 6 | 185.69 | 0 | Prss29 |
|  | 2033 | 11 | 187.77 | 0 | Prss28 |
|  | 244 | 121 | 2.02 | 1.06E-80 | Impdh2 |
|  | 1104 | 266 | 4.15 | 0 | Aprt |
|  | 230 | 100 | 2.3 | 8.46E-101 | Ppp1r14b |
|  | 46 | 193 | -4.17 | 1.29E-218 | Adh1 |
|  | 165 | 76 | 2.18 | 9.29E-65 | Bxdc2 |
|  | 212 | 92 | 2.31 | 1.45E-93 | Emg1 |
|  | 154 | 66 | 2.32 | 3.75E-68 | Gnl3l |
|  | 723 | 201 | 3.59 | 0 | Tsr1 |
|  | 138 | 45 | 3.07 | 7.50E-96 | Nop10 |
|  | 129 | 37 | 3.45 | 7.19E-104 | Ftsj3 |
|  | 210 | 499 | -2.38 | 5.53E-275 | Sepp1 |
|  | 482 | 103 | 4.68 | 0 | Serpine2 |
|  | 107 | 32 | 3.32 | 3.21E-82 | Srm |
|  | 63 | 167 | -2.7 | 1.63E-110 | Cyb5r3 |
|  | 157 | 76 | 2.06 | 1.25E-53 | Idh2 |
|  | 115 | 26 | 4.41 | 1.29E-118 | Sdhb |
|  | 100 | 22 | 4.51 | 8.84E-105 | Fh1 |
|  | 276 | 5 | 52.04 | 0 | Tdo2 |
|  | 217 | 50 | 4.36 | 2.43E-222 | Usp1 |
|  | 242 | 47 | 5.11 | 7.21E-282 | Psma4 |
|  | 109 | 0 | 949.48 | 1.91E-292 | Psma7 |
|  | 16 | 132 | -8.33 | 2.44E-232 | Rnf128 |
|  | 106 | 52 | 2.05 | 4.22E-35 | Ugdh |
|  | 433 | 89 | 4.85 | 0 | Nme2 |
| mRNA processing | 41 | 134 | -3.23 | 2.37E-115 | Zfp36l1 |
|  | 276 | 676 | -2.44 | 0 | Srrm2 |
|  | 193 | 78 | 2.48 | 6.36E-98 | Slbp |
|  | 203 | 81 | 2.51 | 8.20E-105 | Nhp2l1 |
|  | 195 | 88 | 2.22 | 1.84E-79 | Snrpd1 |
|  | 129 | 57 | 2.26 | 3.69E-54 | Tra2b |
|  | 247 | 107 | 2.31 | 2.78E-109 | Snrpe |
|  | 282 | 121 | 2.34 | 1.53E-128 | Dhx15 |
|  | 389 | 158 | 2.47 | 2.91E-196 | Sfrs2 |
|  | 106 | 43 | 2.48 | 9.43E-53 | H47 |
|  | 155 | 59 | 2.61 | 4.30E-85 | Snrpa1 |
|  | 125 | 46 | 2.7 | 5.38E-72 | Ptbp1 |
|  | 116 | 41 | 2.84 | 3.82E-72 | Sf3a3 |
|  | 349 | 113 | 3.1 | 2.47E-248 | Snrpd3 |
|  | 198 | 59 | 3.37 | 1.68E-155 | Lsm5 |
|  | 370 | 107 | 3.45 | 2.48E-300 | Hnrnpa1 |
| receptor activity | 60 | 249 | -4.17 | 9.74E-277 | Pgrmc1 |
|  | 136 | 425 | -3.13 | 0 | Antxr2 |
|  | 143 | 392 | -2.7 | 3.97E-271 | Sema5a |
|  | 77 | 186 | -2.38 | 6.67E-104 | Ramp2 |
|  | 127 | 292 | -2.27 | 5.61E-151 | Ptch1 |
|  | 57 | 117 | -2.08 | 4.52E-48 | Scarb1 |
|  | 227 | 104 | 2.18 | 1.56E-89 | Il11ra1 |
|  | 318 | 91 | 3.51 | 8.34E-263 | Il1r1 |
|  | 109 | 24 | 4.51 | 4.39E-114 | Slit3 |
|  | 188 | 64 | 2.96 | 2.15E-125 | Efemp2 |
| ribosome | 1631 | 4900 | -3.03 | 0 | Rps27 |
|  | 604 | 1418 | -2.33 | 0 | Rpl10a |
|  | 3280 | 7692 | -2.33 | 0 | Rps4x |
|  | 1424 | 3218 | -2.27 | 0 | Rpl32 |
|  | 1760 | 3957 | -2.27 | 0 | Rps14 |
|  | 1112 | 2498 | -2.22 | 0 | Rpl13 |
|  | 1023 | 2261 | -2.22 | 0 | Rps5 |
|  | 1983 | 4086 | -2.04 | 0 | Rps9 |
|  | 1602 | 776 | 2.06 | 0 | Rpl11 |
|  | 744 | 358 | 2.08 | 8.87E-266 | Rpl29 |
|  | 164 | 78 | 2.11 | 2.25E-59 | Mrpl52 |
|  | 177 | 78 | 2.26 | 1.66E-74 | Mrpl17 |
|  | 483 | 214 | 2.26 | 2.71E-206 | Rps27l |
|  | 723 | 227 | 3.18 | 0 | Rps18 |
|  | 315 | 92 | 3.45 | 1.49E-255 | Mrps16 |
| signal transduction | 235 | 62 | 3.77 | 1.22E-209 | Gja1 |
|  | 203 | 4 | 53.3 | 0 | Sct |
|  | 307 | 962 | -3.13 | 0 | Igfbp6 |
|  | 46 | 132 | -2.86 | 9.50E-96 | Igfbp5 |
|  | 22 | 225 | -10 | 0 | Angptl7 |
|  | 40 | 395 | -10 | 0 | Fzd6 |
|  | 29 | 183 | -6.25 | 4.45E-279 | Tacstd2 |
|  | 33 | 158 | -4.76 | 6.71E-199 | Ltbp1 |
|  | 32 | 154 | -4.76 | 4.70E-193 | Dgat2 |
|  | 25 | 120 | -4.76 | 6.02E-150 | Tgfbrap1 |
|  | 33 | 135 | -4.17 | 3.82E-150 | Als2cl |
|  | 94 | 381 | -4 | 0 | Sparcl1 |
|  | 31 | 102 | -3.33 | 4.76E-90 | Iqgap1 |
|  | 60 | 159 | -2.7 | 4.48E-105 | Gabbr1 |
|  | 53 | 140 | -2.63 | 1.19E-91 | Ralgds |
|  | 80 | 180 | -2.22 | 7.76E-88 | Meg3 |
|  | 64 | 141 | -2.17 | 1.47E-65 | Abr |
|  | 53 | 112 | -2.13 | 1.06E-47 | Ocrl |
|  | 141 | 52 | 2.7 | 1.81E-81 | Net1 |
|  | 213 | 63 | 3.36 | 6.08E-167 | Rgs19 |
|  | 350 | 98 | 3.56 | 8.62E-295 | Pak1ip1 |
|  | 367 | 81 | 4.55 | 0 | Fzd1 |
|  | 121 | 371 | -3.03 | 4.80E-301 | Ltbp4 |
| stress response | 15 | 139 | -9.09 | 1.01E-253 | Enpp2 |
|  | 30 | 125 | -4.17 | 3.17E-140 | Procr |
|  | 242 | 946 | -3.85 | 0 | Igj |
|  | 45 | 127 | -2.86 | 3.34E-91 | Fcgrt |
|  | 1176 | 3241 | -2.78 | 0 | Igh |
|  | 92 | 217 | -2.38 | 1.28E-117 | Lbp |
|  | 136 | 286 | -2.13 | 3.89E-124 | Igh-6 |
|  | 151 | 73 | 2.09 | 2.85E-53 | Irg1 |
|  | 216 | 27 | 7.94 | 0 | Oas1a |
|  | 518 | 220 | 2.35 | 5.43E-240 | Mif |
|  | 207 | 626 | -3.03 | 0 | Cd24a |
|  | 57 | 284 | -5 | 0 | Plat |
|  | 58 | 219 | -3.85 | 2.44E-225 | Cxcl12 |
|  | 72 | 270 | -3.7 | 4.04E-274 | Atp1b1 |
|  | 124 | 37 | 3.35 | 6.50E-96 | Tfrc |
|  | 160 | 4 | 39.71 | 0 | Ccl2 |
|  | 45 | 159 | -3.57 | 1.39E-151 | Hsd11b2 |
|  | 68 | 136 | -2 | 5.66E-52 | Cst3 |
|  | 815 | 297 | 2.75 | 0 | Serpinh1 |
|  | 1709 | 365 | 4.68 | 0 | Hspa5 |
|  | 314 | 7 | 47.87 | 0 | Ptx3 |
|  | 57 | 125 | -2.17 | 1.30E-56 | Clec2d |
|  | 295 | 146 | 2.01 | 1.75E-96 | Actr3 |
|  | 120 | 59 | 2.03 | 2.19E-39 | Stip1 |
|  | 1076 | 511 | 2.11 | 0 | B2m |
| transcription | 18 | 324 | -16.67 | 0 | Msx1 |
|  | 62 | 327 | -5.26 | 0 | Tsc22d3 |
|  | 185 | 888 | -4.76 | 0 | Txnip |
|  | 34 | 145 | -4.17 | 2.39E-164 | Hbp1 |
|  | 34 | 124 | -3.7 | 5.66E-123 | Tsc22d1 |
|  | 40 | 127 | -3.13 | 2.12E-105 | Tgif1 |
|  | 34 | 105 | -3.03 | 3.85E-83 | Polr3gl |
|  | 36 | 109 | -3.03 | 1.11E-83 | Mbd6 |
|  | 59 | 161 | -2.7 | 7.20E-110 | Sdpr |
|  | 71 | 189 | -2.63 | 1.30E-123 | Arap1 |
|  | 80 | 212 | -2.63 | 4.14E-139 | Id2 |
|  | 40 | 106 | -2.63 | 3.75E-67 | Hivep2 |
|  | 56 | 145 | -2.56 | 6.89E-91 | Smarcd2 |
|  | 74 | 192 | -2.56 | 9.38E-121 | Ptov1 |
|  | 189 | 487 | -2.56 | 0 | Rarres2 |
|  | 505 | 1231 | -2.44 | 0 | Sfrs5 |
|  | 63 | 151 | -2.44 | 1.47E-84 | Gata2 |
|  | 554 | 1300 | -2.33 | 0 | Ddx17 |
|  | 78 | 173 | -2.22 | 4.59E-83 | Mga |
|  | 90 | 199 | -2.22 | 2.18E-95 | Adnp |
|  | 238 | 520 | -2.17 | 5.35E-245 | Hoxa10 |
|  | 160 | 347 | -2.17 | 1.13E-160 | Jund |
|  | 51 | 111 | -2.17 | 6.36E-50 | Fyn |
|  | 299 | 638 | -2.13 | 1.17E-286 | Rbm39 |
|  | 55 | 115 | -2.13 | 4.38E-49 | Aes |
|  | 58 | 120 | -2.08 | 1.16E-49 | Nr2f2 |
|  | 98 | 203 | -2.04 | 1.16E-83 | Nfatc4 |
|  | 106 | 213 | -2 | 8.10E-83 | Eif3k |
|  | 141 | 68 | 2.08 | 2.89E-49 | Bzw1 |
|  | 235 | 113 | 2.09 | 8.62E-84 | Eif4ebp1 |
|  | 129 | 61 | 2.1 | 6.53E-46 | Foxk2 |
|  | 220 | 103 | 2.14 | 4.51E-83 | Eif3c |
|  | 207 | 96 | 2.14 | 2.89E-78 | Nsfl1c |
|  | 125 | 55 | 2.27 | 1.95E-52 | Atad2 |
|  | 1003 | 419 | 2.39 | 0 | Hnrnpab |
|  | 1058 | 422 | 2.51 | 0 | Eif5a |
|  | 118 | 45 | 2.65 | 3.57E-66 | Mcm7 |
|  | 120 | 44 | 2.75 | 3.42E-71 | Eif5 |
|  | 190 | 65 | 2.93 | 6.95E-125 | Fst |
|  | 122 | 39 | 3.11 | 3.66E-86 | Hnrnpd |
|  | 173 | 51 | 3.41 | 1.24E-137 | Tfdp1 |
|  | 218 | 47 | 4.62 | 5.07E-235 | Id1 |
|  | 174 | 22 | 7.83 | 1.24E-262 | Tceal8 |
|  | 358 | 25 | 14.44 | 0 | Cebpb |
|  | 78 | 171 | -2.17 | 5.46E-79 | Tob1 |
|  | 51 | 136 | -2.63 | 1.92E-87 | Nr1h2 |
|  | 744 | 294 | 2.53 | 0 | Calr |
| translation | 172 | 70 | 2.45 | 3.30E-85 | Ssr3 |
|  | 76 | 211 | -2.78 | 1.10E-147 | Tpr |
|  | 118 | 253 | -2.13 | 2.09E-113 | Paip2 |
|  | 141 | 67 | 2.09 | 7.89E-50 | Ppp1r15b |
|  | 327 | 125 | 2.62 | 5.08E-182 | Rbm3 |
|  | 122 | 26 | 4.68 | 3.90E-132 | Etf1 |
|  | 228 | 35 | 6.43 | 0 | Eif4e2 |
| transport | 16 | 123 | -7.69 | 6.96E-211 | Steap3 |
|  | 58 | 346 | -5.88 | 0 | Clcn1 |
|  | 36 | 201 | -5.56 | 4.80E-281 | Slc2a3 |
|  | 59 | 221 | -3.7 | 7.48E-224 | Selenbp1 |
|  | 39 | 143 | -3.7 | 9.35E-142 | Scara5 |
|  | 64 | 224 | -3.45 | 2.69E-211 | Slc25a36 |
|  | 67 | 223 | -3.33 | 6.44E-197 | Slc39a4 |
|  | 35 | 113 | -3.23 | 2.60E-96 | Slc1a1 |
|  | 70 | 220 | -3.13 | 5.36E-182 | Atp6v0b |
|  | 99 | 293 | -2.94 | 1.63E-225 | Vps28 |
|  | 40 | 104 | -2.63 | 3.73E-67 | Atp6v1f |
|  | 79 | 184 | -2.33 | 3.12E-97 | Slc29a1 |
|  | 53 | 106 | -2 | 5.46E-41 | Atp6v1d |
|  | 612 | 1231 | -2 | 0 | Laptm4a |
|  | 204 | 99 | 2.05 | 9.35E-70 | Copb1 |
|  | 176 | 85 | 2.07 | 4.48E-61 | Mcfd2 |
|  | 112 | 51 | 2.22 | 6.26E-45 | Ipo7 |
|  | 339 | 150 | 2.26 | 9.77E-145 | Tmed10 |
|  | 250 | 107 | 2.34 | 3.20E-114 | Atp5f1 |
|  | 113 | 47 | 2.42 | 1.49E-53 | Rangrf |
|  | 127 | 49 | 2.57 | 3.09E-67 | Sec13 |
|  | 147 | 54 | 2.7 | 3.35E-85 | Ndufb4 |
|  | 390 | 139 | 2.81 | 2.47E-242 | Txn1 |
|  | 281 | 92 | 3.07 | 6.55E-197 | Tmed9 |
|  | 154 | 46 | 3.31 | 4.42E-118 | Rtn3 |
|  | 188 | 55 | 3.43 | 9.46E-151 | Sec61g |
|  | 463 | 134 | 3.45 | 0 | Sec61b |
|  | 152 | 42 | 3.61 | 1.02E-128 | Txnl1 |
|  | 177 | 45 | 3.94 | 1.78E-164 | Pcp4l1 |
|  | 182 | 40 | 4.56 | 2.41E-193 | Fxyd5 |
|  | 777 | 158 | 4.91 | 0 | Tmed3 |
|  | 170 | 34 | 5.03 | 5.24E-195 | Ndufb6 |
|  | 201 | 37 | 5.44 | 6.40E-245 | Slc25a5 |
|  | 127 | 21 | 6.04 | 1.63E-165 | Timm17a |
|  | 163 | 16 | 10.09 | 1.73E-274 | Kpna2 |
| tRNA synthetase | 105 | 50 | 2.09 | 1.03E-36 | Mars |
|  | 165 | 37 | 4.5 | 3.99E-173 | Nars |
|  | 125 | 23 | 5.46 | 7.57E-152 | Tars |
| unkown | 3 | 373 | -100 | 0 | Calb1 |
|  | 4 | 371 | -100 | 0 | 9930023K05Rik |
|  | 3 | 226 | -100 | 0 | AW011956 |
|  | 6 | 288 | -50 | 0 | Thrsp |
|  | 7 | 116 | -16.67 | 1.42E-262 | Fam83a |
|  | 8 | 125 | -14.29 | 1.81E-275 | Pdzk1ip1 |
|  | 12 | 156 | -14.29 | 0 | OTTMUSG00000002043 |
|  | 11 | 142 | -12.5 | 5.04E-296 | Gm967 |
|  | 11 | 105 | -10 | 1.71E-199 | Pik3ip1 |
|  | 25 | 144 | -5.88 | 1.95E-208 | Lrrc17 |
|  | 26 | 139 | -5.26 | 7.45E-189 | LOC629446 |
|  | 36 | 183 | -5.26 | 1.66E-244 | Ilvbl |
|  | 26 | 113 | -4.35 | 1.84E-131 | Arid1b |
|  | 27 | 118 | -4.35 | 6.04E-137 | 4833420G17Rik |
|  | 30 | 118 | -4 | 3.83E-125 | Tspan1 |
|  | 59 | 231 | -3.85 | 5.63E-244 | Mpzl1 |
|  | 27 | 105 | -3.85 | 5.57E-109 | Pink1 |
|  | 28 | 107 | -3.85 | 8.98E-111 | Phldb1 |
|  | 45 | 172 | -3.85 | 1.94E-177 | Hddc2 |
|  | 36 | 134 | -3.7 | 9.99E-132 | Flcn |
|  | 43 | 154 | -3.57 | 3.06E-147 | Wbp2 |
|  | 31 | 109 | -3.57 | 2.91E-102 | Cpe |
|  | 29 | 101 | -3.45 | 6.58E-93 | Trim41 |
|  | 38 | 129 | -3.33 | 5.77E-116 | Plekhh2 |
|  | 116 | 389 | -3.33 | 0 | Leng8 |
|  | 533 | 1747 | -3.23 | 0 | 2310043N10Rik |
|  | 55 | 178 | -3.23 | 1.34E-153 | Bat2l |
|  | 65 | 211 | -3.23 | 1.66E-181 | LOC676142 |
|  | 34 | 109 | -3.23 | 1.04E-90 | Fam173a |
|  | 38 | 117 | -3.03 | 7.62E-93 | 2810008D09Rik |
|  | 47 | 144 | -3.03 | 8.23E-115 | Nelf |
|  | 87 | 262 | -3.03 | 2.60E-204 | 2900092E17Rik |
|  | 143 | 427 | -3.03 | 0 | Dtx3 |
|  | 115 | 342 | -2.94 | 1.56E-265 | LOC100042464 |
|  | 418 | 1204 | -2.86 | 0 | Gas5 |
|  | 60 | 170 | -2.86 | 3.73E-123 | Ypel2 |
|  | 154 | 425 | -2.78 | 1.61E-297 | Frmd6 |
|  | 49 | 129 | -2.63 | 1.36E-84 | Trim47 |
|  | 64 | 169 | -2.63 | 6.50E-110 | Vps52 |
|  | 45 | 118 | -2.63 | 5.49E-76 | Sidt2 |
|  | 41 | 109 | -2.63 | 1.71E-69 | Them2 |
|  | 79 | 206 | -2.63 | 6.74E-131 | 0610010O12Rik |
|  | 45 | 116 | -2.56 | 3.13E-72 | Zc3h7b |
|  | 245 | 628 | -2.56 | 0 | Ifi27l1 |
|  | 216 | 548 | -2.56 | 0 | Itm2b |
|  | 50 | 127 | -2.56 | 2.86E-77 | Grcc10 |
|  | 66 | 165 | -2.5 | 2.36E-99 | Nudt4 |
|  | 53 | 130 | -2.5 | 3.37E-76 | Fam102b |
|  | 93 | 225 | -2.44 | 3.34E-127 | Tmem165 |
|  | 76 | 183 | -2.38 | 5.63E-102 | Crip2 |
|  | 65 | 156 | -2.38 | 9.63E-86 | Ddrgk1 |
|  | 68 | 158 | -2.33 | 3.28E-83 | Tmem181 |
|  | 275 | 636 | -2.33 | 0 | Hoxa11as |
|  | 91 | 206 | -2.27 | 4.34E-104 | 2410006H16Rik |
|  | 51 | 115 | -2.27 | 9.87E-57 | Twsg1 |
|  | 70 | 158 | -2.27 | 2.74E-78 | Frag1 |
|  | 46 | 104 | -2.27 | 2.60E-50 | Wbp1 |
|  | 95 | 213 | -2.27 | 2.40E-105 | Mbnl2 |
|  | 69 | 156 | -2.22 | 3.76E-76 | 2210013O21Rik |
|  | 124 | 278 | -2.22 | 7.76E-137 | Trim35 |
|  | 214 | 479 | -2.22 | 1.04E-235 | Tmem59 |
|  | 48 | 108 | -2.22 | 1.24E-51 | Trafd1 |
|  | 82 | 182 | -2.22 | 8.38E-88 | ENSMUSG00000074747 |
|  | 51 | 113 | -2.22 | 1.73E-53 | Trim44 |
|  | 68 | 150 | -2.17 | 1.98E-70 | Ankrd13a |
|  | 49 | 108 | -2.17 | 4.62E-50 | Tes |
|  | 52 | 113 | -2.17 | 4.59E-52 | Ankrd10 |
|  | 47 | 101 | -2.17 | 1.89E-45 | Rnf216 |
|  | 115 | 248 | -2.17 | 3.00E-113 | Chchd10 |
|  | 62 | 132 | -2.13 | 1.85E-58 | 6820431F20Rik |
|  | 53 | 112 | -2.13 | 3.38E-48 | Emx2 |
|  | 67 | 142 | -2.13 | 1.04E-60 | Pcdhga12 |
|  | 126 | 264 | -2.08 | 2.39E-113 | Zc3h11a |
|  | 57 | 118 | -2.08 | 7.90E-49 | Apcdd1 |
|  | 89 | 182 | -2.04 | 2.47E-72 | Wdfy3 |
|  | 58 | 118 | -2.04 | 2.23E-46 | Fam62b |
|  | 63 | 128 | -2.04 | 4.48E-50 | Iqwd1 |
|  | 74 | 149 | -2.04 | 1.45E-58 | 2500003M10Rik |
|  | 76 | 154 | -2 | 1.24E-59 | Fam38a |
|  | 55 | 111 | -2 | 8.24E-43 | OTTMUSG00000016611 |
|  | 51 | 103 | -2 | 3.61E-39 | Hipk1 |
|  | 59 | 119 | -2 | 2.69E-45 | Anxa11 |
|  | 156 | 77 | 2.02 | 8.73E-51 | Dnaja1 |
|  | 1562 | 766 | 2.04 | 0 | Plac8 |
|  | 152 | 73 | 2.08 | 2.25E-53 | Wbp5 |
|  | 113 | 54 | 2.1 | 4.88E-40 | Esd |
|  | 248 | 117 | 2.12 | 1.03E-91 | Tmem208 |
|  | 104 | 49 | 2.13 | 3.89E-38 | Cops4 |
|  | 345 | 158 | 2.19 | 2.57E-137 | Ssr4 |
|  | 136 | 62 | 2.19 | 1.84E-53 | Slmo2 |
|  | 114 | 52 | 2.2 | 5.84E-45 | Chchd1 |
|  | 169 | 75 | 2.24 | 6.48E-70 | Cops6 |
|  | 307 | 137 | 2.24 | 1.28E-128 | Npm3 |
|  | 138 | 61 | 2.26 | 1.54E-57 | Ppid |
|  | 245 | 101 | 2.42 | 1.42E-118 | Surf4 |
|  | 201 | 79 | 2.54 | 3.75E-106 | Sssca1 |
|  | 274 | 108 | 2.55 | 1.33E-145 | Srp54a |
|  | 235 | 88 | 2.66 | 7.50E-134 | Ly6c1 |
|  | 2122 | 735 | 2.89 | 0 | Spink3 |
|  | 746 | 257 | 2.91 | 0 | 1810022K09Rik |
|  | 289 | 97 | 2.99 | 3.47E-196 | Tdpoz1 |
|  | 141 | 46 | 3.06 | 5.36E-98 | Mtap7d1 |
|  | 540 | 167 | 3.23 | 0 | Ostc |
|  | 102 | 30 | 3.37 | 1.78E-79 | Nlrp12 |
|  | 103 | 29 | 3.54 | 6.75E-85 | 4632417K18Rik |
|  | 227 | 62 | 3.67 | 5.85E-197 | Lrrc59 |
|  | 140 | 38 | 3.73 | 5.47E-123 | Wdr46 |
|  | 251 | 65 | 3.89 | 2.04E-231 | Isg15 |
|  | 114 | 29 | 4 | 2.50E-107 | Dnajb1 |
|  | 896 | 187 | 4.79 | 0 | Armet |
|  | 492 | 102 | 4.82 | 0 | Ly6a |
|  | 101 | 20 | 5.15 | 6.27E-117 | Ifit3 |
|  | 115 | 21 | 5.49 | 1.31E-139 | Abcf2 |
|  | 1695 | 308 | 5.51 | 0 | Ran |
|  | 283 | 50 | 5.72 | 0 | Creld2 |
|  | 116 | 17 | 6.99 | 2.65E-164 | Commd2 |
|  | 890 | 108 | 8.26 | 0 | Nme1 |
|  | 131 | 9 | 15.11 | 7.64E-252 | Prap1 |
|  | 114 | 6 | 18.4 | 5.74E-232 | 2810417H13Rik |
|  | 183 | 0 | 397.26 | 0 | Dio3 |
