# Supplementary material for: The Integrative Analysis of microRNA and mRNA Expression in Mouse Uterus under Delayed Implantation and Activation
Source: PLoS One. 2010 Nov 29;5(11):e15513. doi: 10.1371/journal.pone.0015513 (PMC2993968; doi:10.1371/journal.pone.0015513)
Supplement: Table S4 — The differentially expressed miRNAs and their corresponding coherent target mRNAs during delayed implantation. (DOC) [file pone.0015513.s004.doc]

Table S4 The differentially expressed miRNAs and their corresponding coherent target mRNAs during delayed implantation

| miRNA name | miRNA folds | Gene | mRNA folds | Software |
| --- | --- | --- | --- | --- |
|  | (Activation/Delay) |  | (Activation/Delay) |  |
| miR-92a | 1.93 | Pcmtd1 | 0.26 | PITA |
| miR-92a | 1.93 | Tgif1 | 0.32 | PITA |
| miR-92a | 1.93 | Nelf | 0.33 | PITA |
| miR-92a | 1.93 | Baz2a | 0.35 | PITA |
| miR-805 | 3.71 | Ptch1 | 0.44 | targetscan |
| miR-805 | 3.71 | Ccnl2 | 0.46 | targetscan |
| miR-805 | 3.71 | Fyn | 0.46 | PITA |
| miR-7a | 3.42 | Fam83a | 0.06 | targetscan |
| miR-7a | 3.42 | Nr1h2 | 0.38 | targetscan |
| miR-7a | 3.42 | Ctdsp2 | 0.39 | targetscan |
| miR-7a | 3.42 | Rnf20 | 0.44 | targetscan |
| miR-429 | 0.44 | Ckap4 | 5.57 | PITA |
| miR-429 | 0.44 | Taok1 | 2.58 | PITA |
| miR-429 | 0.44 | Hspa9 | 2.52 | PITA |
| miR-429 | 0.44 | Mcfd2 | 2.07 | targetscan |
| miR-33 | 1.71 | Atp1b1 | 0.27 | targetscan |
| miR-33 | 1.71 | Zfp36l1 | 0.31 | targetscan |
| miR-33 | 1.71 | Abca1 | 0.42 | PITA,targetscan |
| miR-33 | 1.71 | Spin1 | 0.46 | PITA,targetscan |
| miR-33 | 1.71 | Hipk1 | 0.50 | PITA |
| miR-322 | 0.60 | Dio3 | 397.26 | PITA |
| miR-322 | 0.60 | Tceal8 | 7.83 | PITA |
| miR-322 | 0.60 | Rtn3 | 3.31 | PITA |
| miR-31 | 0.50 | Tfrc | 3.35 | PITA,targetscan |
| miR-31 | 0.50 | Eif5 | 2.75 | PITA,targetscan |
| miR-31 | 0.50 | Taok1 | 2.58 | PITA |
| miR-31 | 0.50 | Slbp | 2.48 | PITA |
| miR-31 | 0.50 | Tmed10 | 2.26 | PITA,targetscan |
| miR-29c | 0.54 | Ptx3 | 47.87 | PITA |
| miR-29c | 0.54 | Eif4e2 | 6.43 | PITA |
| miR-29c | 0.54 | Col3a1 | 4.90 | PITA |
| miR-29c | 0.54 | Lox | 3.24 | PITA |
| miR-29c | 0.54 | Morf4l2 | 2.54 | PITA |
| miR-29c | 0.54 | Col4a1 | 2.11 | PITA,targetscan |
| miR-298 | 2.53 | Slc2a3 | 0.18 | PITA |
| miR-298 | 2.53 | Ltbp1 | 0.21 | targetscan |
| miR-298 | 2.53 | Arid1b | 0.23 | PITA |
| miR-298 | 2.53 | Trim41 | 0.29 | PITA,targetscan |
| miR-298 | 2.53 | Leng8 | 0.30 | PITA |
| miR-298 | 2.53 | Ddah2 | 0.31 | PITA |
| miR-298 | 2.53 | Mbd6 | 0.33 | PITA,targetscan |
| miR-298 | 2.53 | Rev3l | 0.34 | PITA |
| miR-298 | 2.53 | Ctdsp2 | 0.39 | targetscan |
| miR-298 | 2.53 | Itm2b | 0.39 | PITA |
| miR-298 | 2.53 | Hoxa10 | 0.46 | PITA |
| miR-298 | 2.53 | Ccnl2 | 0.46 | targetscan |
| miR-23b | 0.51 | Pdia6 | 4.47 | PITA,targetscan |
| miR-23b | 0.51 | Tfrc | 3.35 | PITA |
| miR-23b | 0.51 | Top2b | 2.71 | PITA |
| miR-23b | 0.51 | Tgfbr2 | 2.40 | PITA |
| miR-23b | 0.51 | Mcfd2 | 2.07 | PITA |
| miR-23b | 0.51 | Actr3 | 2.01 | PITA |
| miR-23a | 0.59 | Hmgb2 | 7.72 | targetscan |
| miR-23a | 0.59 | Pdia6 | 4.47 | PITA |
| miR-23a | 0.59 | Tfrc | 3.35 | PITA |
| miR-23a | 0.59 | Ube2d3 | 2.97 | targetscan |
| miR-23a | 0.59 | Top2b | 2.71 | PITA |
| miR-23a | 0.59 | Tgfbr2 | 2.40 | PITA,targetscan |
| miR-23a | 0.59 | Dhx15 | 2.34 | targetscan |
| miR-23a | 0.59 | Col4a1 | 2.11 | targetscan |
| miR-23a | 0.59 | Mcfd2 | 2.07 | PITA,targetscan |
| miR-23a | 0.59 | Actr3 | 2.01 | PITA |
| miR-214 | 0.61 | Dio3 | 397.26 | PITA |
| miR-214 | 0.61 | Srm | 3.32 | PITA |
| miR-214 | 0.61 | Taok1 | 2.58 | targetscan |
| miR-214 | 0.61 | Mapk1ip1l | 2.49 | PITA,targetscan |
| miR-21* | 2.91 | Calb1 | 0.01 | targetscan |
| miR-21* | 2.91 | Pls3 | 0.18 | targetscan |
| miR-21* | 2.91 | Hbp1 | 0.24 | targetscan |
| miR-21* | 2.91 | Mpzl1 | 0.26 | targetscan |
| miR-21* | 2.91 | Cpne3 | 0.29 | targetscan |
| miR-21* | 2.91 | Slc1a1 | 0.31 | targetscan |
| miR-21* | 2.91 | Igf1 | 0.35 | targetscan |
| miR-21* | 2.91 | Atp6v1f | 0.38 | targetscan |
| miR-21* | 2.91 | Hp1bp3 | 0.41 | targetscan |
| miR-21* | 2.91 | Dctn2 | 0.44 | targetscan |
| miR-21* | 2.91 | Spin1 | 0.46 | targetscan |
| miR-21* | 2.91 | Zc3h11a | 0.48 | targetscan |
| miR-200b | 0.58 | Ptbp1 | 2.70 | targetscan |
| miR-200b | 0.58 | Hspa9 | 2.52 | targetscan |
| miR-200b | 0.58 | Surf4 | 2.42 | targetscan |
| miR-200a | 0.57 | Gja1 | 3.77 | PITA |
| miR-200a | 0.57 | Tfrc | 3.35 | PITA |
| miR-200a | 0.57 | Tgfbr2 | 2.40 | PITA |
| miR-200a | 0.57 | Brd4 | 2.11 | targetscan |
| miR-196b | 0.62 | Col14a1 | 3.42 | PITA |
| miR-196b | 0.62 | Calm1 | 2.10 | PITA |
| miR-196b | 0.62 | Ppp1r15b | 2.09 | targetscan |
| miR-16 | 0.59 | Tceal8 | 7.83 | PITA |
| miR-16 | 0.59 | Rtn3 | 3.31 | PITA |
| miR-16 | 0.59 | Ccnd3 | 2.37 | targetscan |
| miR-15b | 0.62 | Tceal8 | 7.83 | PITA |
| miR-15b | 0.62 | Pdap1 | 3.55 | targetscan |
| miR-15b | 0.62 | Rtn3 | 3.31 | PITA |
| miR-15b | 0.62 | Mapk1ip1l | 2.49 | PITA |
| miR-15b | 0.62 | Brd4 | 2.11 | targetscan |
| miR-15a | 0.58 | Tceal8 | 7.83 | PITA |
| miR-15a | 0.58 | Pdia6 | 4.47 | targetscan |
| miR-15a | 0.58 | Hnrnpa1 | 3.45 | targetscan |
| miR-15a | 0.58 | Rtn3 | 3.31 | PITA |
| miR-15a | 0.58 | Pim3 | 3.10 | targetscan |
| miR-146b | 3.52 | Pls3 | 0.18 | PITA |
| miR-146b | 3.52 | Mbnl2 | 0.44 | PITA |
| miR-145 | 0.39 | H2afx | 10.55 | PITA,targetscan |
| miR-145 | 0.39 | Tfrc | 3.35 | targetscan |
| miR-145 | 0.39 | Lox | 3.24 | targetscan |
| miR-145 | 0.39 | Ube2d3 | 2.97 | PITA |
| miR-145 | 0.39 | Net1 | 2.70 | targetscan |
| miR-145 | 0.39 | Rbm3 | 2.62 | targetscan |
| miR-145 | 0.39 | Ube2n | 2.61 | PITA |
| miR-145 | 0.39 | Taok1 | 2.58 | PITA |
| miR-145 | 0.39 | Tgfbr2 | 2.40 | PITA,targetscan |
| miR-145 | 0.39 | Actr3 | 2.01 | PITA |
| miR-138 | 0.47 | Tsr1 | 3.59 | PITA |
| miR-138 | 0.47 | Taok1 | 2.58 | PITA |
| miR-138 | 0.47 | Ccnd3 | 2.37 | targetscan |
| miR-138 | 0.47 | Itga5 | 2.20 | PITA |
| miR-138 | 0.47 | Eif4ebp1 | 2.09 | targetscan |
| miR-134 | 2.60 | Igf1 | 0.35 | PITA |
| miR-134 | 2.60 | Antxr1 | 0.43 | targetscan |
