# Supplementary material for: The Integrative Analysis of microRNA and mRNA Expression in Mouse Uterus under Delayed Implantation and Activation
Source: PLoS One. 2010 Nov 29;5(11):e15513. doi: 10.1371/journal.pone.0015513 (PMC2993968; doi:10.1371/journal.pone.0015513)
Supplement: Table S5 — Primers used in this study. (DOC) [file pone.0015513.s005.doc]

Table S5 Primers used in this study

| Primers | Sequences |
| --- | --- |
| Tmem55a for cloning | Pf: 5'-GAGTCTAGAATCGTCACCTAAAACTTCGC-3'  Pr: 5'-ATAGGCCGGCCTAGCATATTTTCAAAGGTAC-3' |
| TIMP3 for cloning | Pf: 5'-GAATCTAGAGCAACCTGCCCGTCTGTA-3'  Pr: 5'-AAAGGCCGGCCGAAGGCCCTAACCTAACA-3' |
| Smad7 for cloning | Pf: 5'-CGGTCTAGACCCAGTCACAGTATTGCTACC-3'  Pr: 5'-TAAGGCCGGCCTTCTTTTAAGGAGTCCTTTC-3' |
| Klf9 for cloning | Pf: 5'-GCTCTAGATCCGACTTCCAATACGACA-3'  Pr: 5'-TATGGCCGGCCAAGCCACATACAGATTTACTCC-3' |
| Gatm for cloning | Pf: 5'-GCTCTAGAAGTTATTTACGATTTGGCT-3'  Pr: 5'-TATGGCCGGCCGATATTGAACATCAACCTTT-3' |
| Dnajb9 for cloning | Pf: 5'-GCTCTAGAGCCCTGACTTGGGTTA-3'  Pr: 5'-TATGGCCGGCCGAAATTACTGGCTGCTT-3' |
| Tmem55a for real time | Pf: 5'-AGCCAGAAGGCACAAGGGTA-3'  Pr: 5'-ATTCCGATGGTGACATACGC-3' |
| Timp3 for real time | Pf: 5'-CAGTTCAGGAGATGGGTGTCAAG-3'  Pr: 5'-AACAGTAAAGCCAGAGGTTCAGC-3' |
| Smad7 for real time | Pf: 5'-GCCACCGTTCAAACTACTTGC-3'  Pr: 5'-CCAACAATGAATGGCAATAACAAC-3' |
| Smad7 for in situ hybridization | CGGTGCTCAAGAAACTCAA  CCCAGGCTCCAGAAGAAG |
| Ctss for real time | Pf: 5'-GGGAATAAAGGCTGTGGAGG-3'  Pr: 5'-CGTGGCTTTGTAGGGATAGG-3' |
| Mecp2 for real time | Pf: 5'-GAGTCTTCCATACGGTCTGTGC-3'  Pr: 5'-TTCAGTCCCTTCCCGCTTTT-3' |
| Gzma for real time | Pf: 5'-TCAATAAGGAGCCAGAACAACA-3'  Pr: 5'-AGGTAGGTGAAGGATAGCCACA-3' |
| Akr1b7 for real time | Pf: 5'-GTAGGCTACCATCTGGCAATG-3'  Pr: 5'-TACTACATGCTCCTGATGACAAAC-3' |
| Pre-novel-miRNA-1 | Pf: 5'-TAAAGATGTTATGGGGTGTGCTC-3'  Pr: 5'-CAGATTCTTCTTGGGTGTGTTGTA-3' |
| Pre-novel-miRNA-2 | Pf: 5'-CACCCGTCCCGTTCGT-3'  Pr: 5'-CCAGTCTCGACGTTCCCG-3' |
| Pre-novel-miRNA-3 | Pf: 5'-ATGGGGCTTTGGGTGAGG-3'  Pr: 5'-AATGGGCTGGGAGGAAGG-3' |
| Pre-novel-miRNA-4 | Pf: 5'-GGGGACAGAGTATGCGAGAGTA-3'  Pr: 5'-GATGGAAGCAGAATGTTGGAGTA-3' |
| Pre-novel-miRNA-5 | Pf: 5'-CCTGGGAGGAGACGTGGATT-3'  Pr: 5'-GCGAGCTGAACCTGGAACAA-3' |
| Pre-novel-miRNA-6 | Pf: 5'-AGGCTCTCCTAAGGCAGGCA-3'  Pr: 5'-AAGGCCCTTTGGAGGTAAGC-3' |
| Novel-miRNA-1 | Pf: 5'-GTCCATTGGGGTGTGCTCA-3'  P-RT: 5'-GTCGTATCCAGTGCAGGGTCCGAGGTATTCGCACT-  GGATACGACAGGCC-3' |
| Novel-miRNA-2 | Pf: 5'-GTGACCCGTCCCGTTCGT-3'  P-RT: 5'-GTCGTATCCAGTGCAGGGTCCGAGGTATTCGCACT-  GGATACGACTCCGG-3' |
| Novel-miRNA-3 | Pf: 5'-CGCAGGGGAGCTAGGTAGAA-3'  P-RT: 5'-GTCGTATCCAGTGCAGGGTCCGAGGTATTCGCACT-  GGATACGACTGGCT-3' |
| Novel-miRNA-4 | Pf: 5'-GCGATTGGAGTTCATGCAAGT-3'  P-RT: 5'-GTCGTATCCAGTGCAGGGTCCGAGGTATTCGCACT-  GGATACGACAGAAC-3' |
| Novel-miRNA-5 | Pf: 5'-ATCCCTGGGAGGAGACGTG-3'  P-RT: 5'-GTCGTATCCAGTGCAGGGTCCGAGGTATTCGCACT-  GGATACGACGAATC-3' |
| Novel-miRNA-6 | Pf: 5'-CGCTAAGGCAGGCAGACTTC-3'  P-RT: 5'-GTCGTATCCAGTGCAGGGTCCGAGGTATTCGCACT-  GGATACGACACACT-3' |
| General primer | Pr: 5'-CAGTGCAGGGTCCGAGGTAT-3' |
